# Biological Consequences and Assessment Methods Analysis of Fixed Orthodontic Appliances on Oral Epithelial Cells: A Systematic Review

**DOI:** 10.1111/jop.13643

**Published:** 2025-05-19

**Authors:** Francesco Paolo Modugno, Elisabetta Kuhn, Chiara Luisa Bianchi, Letterio Runza, Matteo Pellegrini, Federica Pulicari, Francesco Spadari

**Affiliations:** ^1^ Section of Dentistry, Department of Clinical, Surgical, Diagnostic and Pediatric Sciences University of Pavia Pavia Italy; ^2^ Anatomical Pathology Unit Fondazione IRCCS Cà Granda Ospedale Maggiore Policlinico Milan Italy; ^3^ Department of Biomedical, Surgical and Dental Sciences University of Milan Milan Italy; ^4^ Maxilo‐Facial Surgery and Dental Unit Fondazione IRCCS Cà Granda Ospedale Maggiore Policlinico Milan Italy

**Keywords:** cytology, dentistry, fixed orthodontic appliances, metal, oral mucosa

## Abstract

**Introduction:**

Fixed orthodontic appliances (OAs) expose the oral mucosa to mechanical traumas and metal ions throughout the whole orthodontic therapy. This review aims to understand the cytological and genetic changes consequent to fixed orthodontic therapy, their clinical implications, and how they can be assessed.

**Materials and Methods:**

A comprehensive search was conducted in PubMed (MEDLINE), Scopus, and Web of Science using MeSH terms related to cytology, DNA damage, mutagenicity, and orthodontic appliances. The PICO model and PRISMA guidelines were followed. The risk of bias was assessed using the ROBINS‐I tool, and study quality was evaluated with the NHLBI Quality Assessment Tools. Two independent evaluators assessed the methodological quality of the included studies using the Joanna Briggs Institute (JBI) levels of evidence; inter‐reviewer agreement was measured using Cohen's kappa coefficient (*κ* = 0.80).

**Results:**

Nineteen prospective and cross‐sectional studies were included in the analysis. The findings suggest the presence of higher metal cellular content, as well as cytological changes, nuclear alterations, and cytotoxic and genotoxic effects. Different appliance compositions and treatment durations may influence the biological consequences. The analysis shows a tendency toward regression, especially for nuclear alterations. No dysplastic changes have been observed in any of the studies included.

**Conclusion:**

OAs cause cellular alterations, which tend to be reversible and do not seem to evolve into dysplastic changes. Future research should focus on longitudinal studies with standardized methodologies to better understand the persistence and reversibility of the changes associated with OAs, as well as exploring alternative materials that pose less risk during orthodontic treatment.

**Abbreviations**
BCA: buccal comet assay
CA: cytoplasmic area
CC: condensed chromatin
CS: composite score
DI: damage index
DF: damage frequency
DNA: deoxyribonucleic acid
FOT: fixed orthodontic treatment
KL: karyolysis
KR: karyorrhexis
MN: micronuclei
N/C: nuclear/cytoplasmic ratio
NA: nuclear area
NB: nuclear buds
NHLBI: National Heart, Lung, and Blood Institute
OA: orthodontic appliance
PAP: papanicolaou
PK: pyknosis
PRISMA: Preferred Reporting Items for Systematic Reviews
RR: relative risk
ROBINS‐I: Risk Of Bias in Non‐Randomized Studies–of Interventions
WoS: Web of Science

## 1 Introduction

Orthodontic appliances (OAs) threaten oral epithelial cells through mechanical and biochemical mechanisms. Previous studies have indicated a higher incidence of oral mucosa erosions, ulcerations, and general gingival inflammation in subjects wearing OAs [1, 2]. In vitro studies have demonstrated elevated levels of metal ions in epithelial cells exposed to metallic OAs compared to controls. Commonly released metals include titanium, nickel, chromium, and cobalt [3, 4], which have been linked with lung and prostate cancers. Titanium exhibits the highest biocompatibility, regardless of its potential to cause DNA‐strand breaks and base oxidation. Nickel is notorious for its genotoxic effects and has been convincingly linked to lung and prostate tumors. Chromium (IV) is a recognized carcinogenic agent for the lung and nasal mucosa. Cobalt can disrupt DNA repair mechanisms, leading to DNA damage, protein crosslinking, or chromatid exchange; however, its toxicity is comparatively lower than that of other metals [3].

Spectrometry is a technique employed to analyze the chemical composition of substances, including metals within cells. The sample, once prepared, interacts with electromagnetic radiation from various sources [4]. The remaining radiation is then detected, measured, and analyzed, providing insights into the sample's composition and concentration of specific components. Spectrometry encompasses various techniques that differ based on the used radiation, sample characteristics, and desired information. Some common techniques include mass spectrometry (which measures the mass‐to‐charge ratio of ions) and atomic absorption spectrometry (which quantifies the absorption of light by free atoms). The first method showed a wider range of linearity in calibration curves, being able to identify lower levels of metal ions [5, 6].

Cytology is a valuable sample for evaluating tissue and cellular characteristics without the need for a biopsy. Dysplastic conditions show nuclear atypia such as high nuclear/cytoplasmic ratio (N/C), nuclear hyperchromia, curved chromatic bands, prominent nucleoli, and atypical mitoses, often associated with acanthosis and sub‐epithelial inflammatory infiltrate [7]. Developed for exfoliative cytology of the uterine cervix, the Papanicolau (Pap) classification system remains the most frequently applied to oral cytopathology, although it is controversial in the absence of a specific oral cytology classification. In summary, it distinguishes five classes: Class I, absence of abnormal/atypical cells; Class II, atypical cells, but not evidence for malignancy; Class III, suggestive but not conclusive for malignancy; Class IV, strongly suggestive of malignancy; and Class V, positive for malignancy [8]. Micronuclei (MN), which are small and round extranuclear structures formed through altered chromosome segregation during mitosis, indicate chromosomal instability and can lead to the formation of fragmented chromosomes. They are also generated by exposure to genotoxic agents. The MN test has high sensitivity in evaluating cytogenetic damage, particularly in children exposed to environmental genotoxic factors [9]. Cell viability can be assessed using the trypan blue dye exclusion assay: live cells possessing an intact cellular membrane exclude the dye, resulting in unstained cells. Conversely, dead cells absorb the dye, leading to staining [10].

The buccal comet assay (BCA) is used to assess DNA damage. Once the sample is processed, it is subjected to electrophoresis under alkaline conditions, causing DNA migration from cells. DNA containing breakpoints migrates toward the anode, forming comet‐like structures visible under fluorescence microscopy [11, 12]. The head of the comet consists of intact, undamaged DNA that remains in its original position, while the tail consists of fragmented DNA pieces that migrated during the electrophoresis. A higher head diameter typically indicates less DNA damage, while longer tails indicate more significant damage. Automated analysis provides measurements such as tail length and tail DNA percentage, with the latter showing a linear correlation with damage extent. The tail moment, a product of these parameters, is sometimes questioned due to its nonlinear relationship with damage extent. Alternatively, visual assessment categorizes comets into five classes, ranging from tailless (0) to tail‐growing (1–4). The evaluation of 100 comets in a sample, with scores summed to obtain a final value between 0 and 400, provides a visual means of assessing DNA damage [12].

Numerous studies have examined cellular alterations due to the presence of OAs, both in vitro and in vivo. To date, the main published reviews in the literature have different issues: they use different types of samples (e.g., saliva, hair, oral mucosa cells) [13], they consider a limited number of studies [14, 15], or they lack studies performing long‐term assessments [13, 14, 15].

This review aims to evaluate non‐dysplastic and dysplastic alterations in oral epithelial cells associated with fixed orthodontic treatment (FOT), with a particular focus on long‐term studies available to date, to determine the reversibility of these effects.

## 2 Materials and Methods

### 2.1 Focused Question

What are the alterations of the oral epithelial cells associated with FOT? Are the alterations reversible? Does orthodontic treatment predispose to neoplastic transformation?

### 2.2 Eligibility Criteria

The inclusion criteria considered for this review were (I) study design—prospective observational cohort studies and case–control studies; (II) study population—human participants of any age undergoing FOT; (III) exposure—oral epithelial cell exposition to the fixed OA; and (IV) outcome—evidence of oral epithelial cellular alterations and risk factors due to the presence of the fixed OA. The analysis was limited to studies that satisfied all the inclusion criteria, while the exclusion criteria comprised the following aspects: (I) abstracts of articles published in non‐English languages; (II) duplicate studies; (III) studies lacking detailed information on cytologic and genetic alterations in the presence of fixed OA; (IV) ex vivo or experimental animal studies; (V) studies without ethics committee approval; and (VI) narrative, systematic, or meta‐analysis reviews.

### 2.3 Search Strategy

A three‐stage search process was executed following the methodology described by the Joanna Briggs Institute (JBI) for systematic reviews. Initially, preliminary and restricted exploration was carried out using PubMed (MEDLINE), Scopus, and the Web of Science (WoS). Subsequently, the relevant terminology was extracted from the articles to formulate an all‐encompassing research strategy. Finally, the reference lists of all articles were searched to identify any additional pertinent research [16].

The PICO model was used to conduct this review through a literature search of the PubMed (MEDLINE), Scopus, and WoS electronic databases, founded on the following four aspects: population (participants of any age undergoing FOT), intervention (oral epithelial cell exposition to fixed OA), comparison (subjects not undergoing FOT or patients after debonding), and outcomes (evidence of cellular alterations, with their clinical implications). This systematic review was conducted following the Preferred Reporting Items for Systematic Reviews and Meta‐analyses (PRISMA) Statement guidelines, as depicted in Table S1 [17]. Table S2 shows the search strategy used in this review.

### 2.4 Research

Electronic exploration was performed using PubMed (MEDLINE), Scopus, and WoS. Articles published between 2000 and 2024 were included. Data were extracted between December 2023 and March 2024, and a final search was conducted on March 5, 2024. Any duplicate entries in the databases were identified and subsequently eliminated using the EndNote Web reference manager software (version 20) by Clarivate Analytics, based in Philadelphia, PA, USA.

The search was conducted by two reviewers (E.K. and C.L.B.), who worked independently and in duplicate to ensure reliability and reduce selection bias. Each reviewer screened the titles and abstracts of the articles separately. In cases of disagreement, a third reviewer (F.S.) was consulted to reach a consensus. If further clarification was needed, additional reviewers (F.P.M., L.R., M.P., and F.P.) were involved in the decision‐making process.

Consensus among the reviewers was reached by following PRISMA guidelines for study selection. Any disparities that emerged during the review were resolved through the intervention of a third reviewer (F.S.). For complex cases, four additional reviewers (F.P.M., L.R., M.P., and F.P.) were consulted. The initial phase of screening involved the assessment of article titles and abstracts, excluding irrelevant studies. Subsequently, the relevant articles underwent a comprehensive evaluation involving thoroughly examining their full content.

To assess the consistency and agreement between the reviewers during the study selection process, inter‐rater reliability was measured using Cohen's kappa coefficient [18]. A preliminary calibration phase was performed before the screening process, where the reviewers assessed 50 random subset of studies and discussed discrepancies to align their criteria. The Cohen's kappa value obtained was 0.80, indicating a substantial level of agreement according to the Fleiss Guide [19], reinforcing the robustness of the selection methodology.

The present protocol was registered on the PROSPERO platform (CRD42024535897), available at: https://www.crd.york.ac.uk/prospero/display_record.php?RecordID=535897.

### 2.5 Data Extraction

The data extraction process was also conducted in duplicate and independently by the two primary reviewers. Discrepancies were resolved through discussion or, if necessary, by consulting a third reviewer. The extracted data were recorded in a structured spreadsheet and included study characteristics, study design, number of participants, OA composition, sample collection technique, type of analysis conducted on the sample, outcome measured, and key findings.

Following the review of the publications, a spreadsheet was generated and subsequently updated sequentially. The collected data were organized into tables, which provided a structured presentation of the information: the name of the first author of the article and the year of publication, the type of the OA, the factors investigated, analyses performed on the samples, and the results of this analysis.

### 2.6 Quality Assessment of Included Studies

In this study, the potential for bias in clinical studies was assessed through a qualitative analysis using the National Heart, Lung, and Blood Institute (NHLBI) (Bethesda, Maryland, United States) Quality Assessment Tools. This approach enabled a comprehensive and methodical evaluation of the quality and potential biases within the included studies, aiming to establish the dependability and credibility of the results [20].

## 3 Results

**FIGURE 1:**
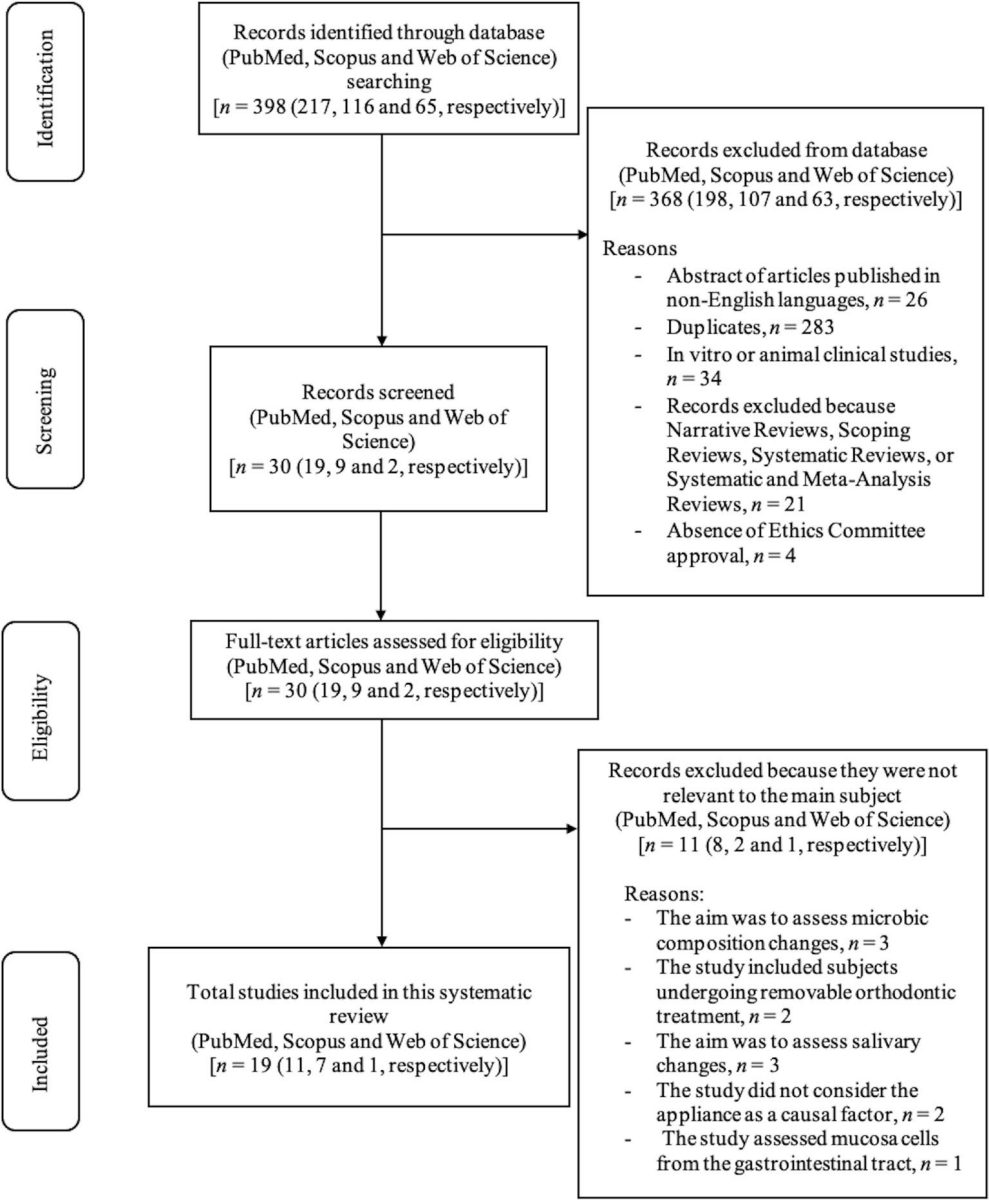
Flowchart of the review process.

An initial search has been conducted using Medical Subject Headings, resulting in 398 articles. A total of 368 articles have been excluded for several reasons: abstracts published in a non‐English language, duplicates, full‐text content not corresponding to the abstract, in vitro or animal clinical studies, review studies, and absence of the approval of the Ethics Committee. A total of 30 articles were then assessed based on their abstracts. Nineteen of them have been considered suitable since they respect the eligibility criteria; therefore, they were included in the in‐depth analysis. The remaining 11 articles have been excluded because they were not relevant to our main goal: they assessed microbic and salivary changes, they considered patients with removable OAs, and/or they did not consider OA as a causal factor or oral cells as a target for the assessment. Figure 1 shows a flowchart of the review process. Table S3 displays the research papers not considered in this analysis because they were not relevant to the main subject, with the explanations for their exclusion [21, 22, 23, 24, 25, 26, 27, 28, 29, 30, 31].

The studies included in this systematic review were all prospective or cross‐sectional observational cohort and case–control studies [32, 33, 34, 35, 36, 37, 38, 39, 40, 41, 42, 43, 44, 45, 46, 47, 48, 49, 50].

### 3.1 Risk of Bias

The assessment of bias risk in the articles included in this review was conducted using the Risk Of Bias In Non‐Randomized Studies—of Interventions (ROBINS‐I) assessment tool (version 19, September 2016). Criteria for judging the risk of bias in the ROBINS‐I assessment tool [51] are outlined in Table S4. The outcomes of this assessment are shown in Table S5, revealing a low risk of bias. The NHLBI Quality Assessment Tool for Observational Cohort Studies is presented in Table S6.

Table 1 presents the baseline characteristics of patients, the study design, the appliance composition, and the sample collection zone of the studies included in this review. Tables 2, 3, 4, 5 give detailed information about the setup of the studies analyzed in this review, including the division into subgroups (based on OA composition, collection zone, or experimental/control groups), the time points, and the significant findings, with the respective *p* value. The tables are divided based on the aim of the studies included, namely, metal cellular content, cytological changes, nuclear alterations, cytotoxicity, and genotoxicity.

**TABLE 1:** Baseline characteristics of the patients, study design, appliance composition, and sample collection zone of the studies included in this review.

Reference	Number of patients	Appliance composition	Collection zone
Authors	Sex (men:women)		
Year of publication	Age (range or mean ± SD)		
Origin of the research			
Study design			
[32] Natarajan et al. 2010 India Prospective case–control observational study	40 N.R. 14–24	Brackets: stainless steel Bands: stainless steel Archwires: Ni‐Ti alloys	Buccal mucosa
[33] Hafez et al. 2011 Egypt Prospective case–control longitudinal study	46 14:32 21.5 ± 3.3 (control); 20.2 (experimental)	Brackets: titanium or stainless steel Archwire: stainless steel or Ni‐Ti alloys Ligature: metal	Buccal mucosa
[34] Kapadia et al. 2018 India Prospective cohort observational study	80 N.R. 13.5	Brackets: stainless steel Bands: stainless steel Archwires: stainless steel	Buccal mucosa
[35] Faccioni et al. 2003 Cross‐sectional case–control observational study	85 49:36 12–35	Brackets: stainless steel Bands: stainless steel Archwires: Ni‐Ti alloy, stainless steel or Cr‐Cb‐Ni alloy	Buccal mucosa
[36] Amini et al. 2008 India, Iran, and the United Kingdom Cross‐sectional case–control observational study	60 40:20 16–20	Brackets: stainless steel Bands: stainless steel (eight patients) Archwire: stainless steel (24 patients) or Ni‐Ti alloy (six patients)	Buccal mucosa
[37] Fernández‐Miñano et al. 2011 Prospective cohort observational study	15 N.R. 12–16	Group A Brackets: stainless steel Tubes: stainless steel	Buccal mucosa
		Group B Brackets: titanium Bands: titanium	
		Group C Brackets: nickel‐free metal Tubes: nickel‐free metal	
[38] Alsalhi et al. 2019 Saudi Arabia Cross‐sectional observational case–control study	100 N.R. 14–28	N.R.	Buccal mucosa
[39] Sampaio Mei et al. 2013 Brazil Prospective observational cohort study	20 10:10 22.7	Brackets: stainless steel Wire: stainless steel Ties: stainless steel or elastic	Buccal mucosa
[40] Arruda et al. 2011 Brazil Cross‐sectional cohort observational study	20 20:0 17–42	Brackets: metal Bands: metal Archwires: Cr‐Cb alloy	Mucosa contacting the brackets Mucosa contacting the tubes Mucosa not contacting the appliance
[41] Rafighi et al. 2020 Iran Prospective cohort observational study	31 10:21 23.0 ± 4.6	Brackets: stainless steel Bands: etched metal Archwires: Ni‐Ti alloy and stainless steel	Lower lip mucosa
[42] Pereira et al. 2008 Brazil Prospective cohort observational study	21 7:14 7.6–53.7	Brackets: metal and ceramic	Buccal mucosa
[43] Buczko et al. 2017 Prospective cohort observational study	28 N.R. 21–24	Brackets: stainless steel	N.R.
[44] Carrillo‐Novia et al. 2006 México Prospective cohort observational study	18 12:6 10–29	Brackets: metal with Cr‐Cb slots Archwires: Ni‐Cr alloys	N.R.
[45] Francis et al. 2017 Prospective cohort observational study	30 15:15 18–25	N.R.	Buccal mucosa
[46] Cunha et al. 2018 Brazil Prospective cohort observational study	28 11:17 9.25 ± 1.5	Bands: silver and stainless steel Wire: stainless steel Union block: acrylic resin Solders: silver	Buccal mucosa
[47] Gonçalves et al. 2015 Prospective cohort observational study	20 N.D. 7–14	Bands: stainless steel Bars: stainless steel Solders: silver	Buccal mucosa
[48] Angelieri et al. 2011 Brazil Prospective cohort observational study	23 10:13 11–35	Brackets: Ni‐Cr alloy Bands: Ni‐Cr alloy Archwires: Ni‐Ti alloy or stainless steel	Buccal mucosa
[49] Flores‐Bracho et al. 2019 Cross‐sectional case–control observational study	74 N.R. 11–35	Brackets: stainless steel Bands: stainless steel Tubes: stainless steel Archwires: stainless steel	Buccal mucosa
[50] Westphalen et al. 2008 Prospective cohort observational study	20 6:14 16 ± 2.5	Brackets: stainless steel Tubes: stainless steel Archwires: stainless steel	Lower lip mucosa

Abbreviation: N.R., not reported.

**TABLE 2:** Study variables and significant metal cellular content changes observed in the studies included in this review.

Reference	Subgroups	Collection time	Parameter that showed significant changes	
			Significant values	Changes and *p* value^a^
[32] Natarajan et al. 2010	—	T0 (case): after debonding T0 (control): whenever T1 (case): 30 days after debonding T1 (control): 30 days after T0	None	
			—	—
[33] Hafez et al. 2011	Stainless steel (Group I) Stainless steel and Ni‐Ti alloy (Group II) Stainless steel and titanium (Group III) Ni‐Ti alloy and titanium (Group IV) Nothing (control)	T0: Before the installation T1: 3 months after installation T2: 6 months after installation	Cellular nickel content (ng/mL) Case vs. control	
			T0 (case): 0.52 ± 0.32 T1 (case): 0.68 ± 0.28 T2 (case): 0.78 ± 0.19	T0 (case) vs. **T1 (case)**, *p* = 0.000 T0 (case) vs. **T2 (case)**, *p* = 0.000 T1 (case) vs. **T2 (case)**, *p* = 0.001
			Cellular chromium content (ng/mL) Case vs. control	
			T0 (case): 0.31 ± 0.25 T1 (case): 0.41 ± 0.28 T2 (case): 0.58 ± 0.26	T0 (case) vs. **T1 (case)**, *p* = 0.000 T0 (case) vs. **T2 (case)**, *p* = 0.000 T1 (case) vs. **T2 (case)**, *p* = 0.002
			Cellular nickel content (ng/mL) Case vs. case	
			T0 (I): 0.40 ± 0.26 T0 (II): 0.43 ± 0.18 T0 (III): 0.69 ± 0.51 T1 (I): 0.63 ± 0.24 T1 (II): 0.57 ± 0.17 T1 (III): 0.91 ± 0.44 T2 (I): 0.76 ± 0.27 T2 (II): 0.64 ± 0.07	T0 (I) vs. **T1 (I)**, *p* = 0.011 T0 (I) vs. **T2 (I)**, *p* = 0.011 T1 (I) vs. **T2 (I)**, *p* = 0.035 T0 (II) vs. **T1 (II)**, *p* = 0.026 T0 (II) vs. **T2 (II)**, *p* = 0.026 T1 (II) vs. **T2 (II)**, *p* = 0.026 T0 (III) vs. **T1 (III)**, *p* = 0.026
			Cellular chromium content (ng/mL) Case vs. case	
			T0 (I): 0.29 ± 0.08 T0 (II): 0.20 ± 0.16 T0 (III): 0.46 ± 0.50 T1 (I): 0.44 ± 0.13 T1 (II): 0.29 ± 0.12 T1 (III): 0.60 ± 0.53 T2 (I): 0.70 ± 0.25 T2 (II): 0.57 ± 0.14	T0 (I) vs. **T1 (I)**, *p* = 0.011 T0 (I) vs. **T2 (I)**, *p* = 0.011 T1 (I) vs. **T2 (I)**, *p* = 0.011 T0 (II) vs. **T1 (II)**, *p* = 0.026 T0 (II) vs. **T2 (II)**, *p* = 0.026 T1 (II) vs. **T2 (II)**, *p* = 0.026 T0 (III) vs. **T1 (III)**, *p* = 0.026
[34] Kapadia et al. 2018	—	T0: before installation T1: 5 months after installation T2: 10 months after installation T3: 15 months after installation T4: 20 months after installation	Cellular nickel content (ppb)	
			T0: 22 T1: 25.3 T2: 39.1 T3: 49.4 T4: 59.7	T0 vs. **all**, *p* = 0.02 T1 vs. **all**, *p* = 0.02 T2 vs. **all**, *p* = 0.02 T3 vs. **all**, *p* = 0.02 T4 vs. **all**, *p* = 0.02
			Cellular chromium content (ppb)	
			T0: 21.1 T1: 25.3 T2: 34.7 T3: 43.9 T4: 55.4	T0 vs. **all**, *p* = 0.01 T1 vs. **all**, *p* = 0.01 T2 vs. **all**, *p* = 0.01 T3 vs. **all**, *p* = 0.01 T4 vs. **all**, *p* = 0.01
			Cellular zinc content (ppb)	
			T0: 194.6 T1: 252.4 T2: 328.7 T3: 434.2 T4: 547.4	T0 vs. **all**, *p* = 0.02 T1 vs. **all**, *p* = 0.02 T2 vs. **all**, *p* = 0.02 T3 vs. **all**, *p* = 0.02 T4 vs. **all**, *p* = 0.02
[35] Faccioni et al. 2003	Stainless steel, Ni‐Ti alloy or titanium (case) None (control)	T0 (case): from 2 to 4 years after the installation T0 (control): whenever	Cellular nickel content (ng/mL)	
			T0 (control): 0.72 ± 0.63 T0 (case): 2.52 ± 1.76	T0 (control) vs. **T0 (case)**, *p* < 0.0001
			Cellular cobalt content (ng/mL)	
			T0 (control): 0.20 ± 0.09 T0 (case): 0.57 ± 0.40	T0 (control) vs. **T0 (case)**, *p* < 0.0001
[36] Amini et al. 2007	Stainless steel and/or Ni‐Ti alloy (case) None (control)	T0 (case): 16 months after installation T0 (control): whenever	Cellular nickel content (ng/mL)	
			T0 (control): 12.26 ± 12.9 T0 (case): 21.74 ± 11.41	T0 (control) vs. **T0 (case)**, *p* = 0.003
[37] Fernández‐Miñano et al. 2011	Stainless steel (Group I) Titanium (Group II) Nickel‐free metal (Group III)	T0: before installation T1: 30 days after installation	Cellular chromium content (microg/L)	
			T0 (III): 0.00 ± 0.00 T1 (III): 0.34 ± 0.29	T0 (III) vs. **T1 (III)**, *p* < 0.05
			Cellular titanium content (microg/L)	
			T0 (I): 0.98 ± 0.64 T1 (I): 3.04 ± 1.67	T0 (I) vs. **T1 (I)**, *p* < 0.05
			Cellular manganese content (microg/L)	
			T0 (I): 0.32 ± 0.30 T1 (I): 1.08 ± 1.07	The T0 (I) vs. **T1 (I)**, *p* < 0.05
			Cellular iron content (microg/L)	
			T0 (III): 1.95 ± 1.29 T1 (III): 5.36 ± 2.44	T0 (III) vs. T1 **(III)**, *p* < 0.05

^a^ Bold text indicates that the measurement is greater than the other.

**TABLE 3:** Study variables and significant cytomorphological and cytomorphometric changes observed in the studies included in this review.

Reference	Subgroups	Collection time	Significant values	
			Parameter that showed significant changes	Changes and *p* value^a^
[38] Alsalhi et al. 2019	—	T0 (case): 3–6 months T0 (controls): whenever	Number of cells with deviation from normal morphology	
			T0 (case): 8.6 ± 1.7 T0 (control) 4.7 ± 2.05	**T0** (case) vs. T0 (control), *p* < 0.0001
[39] Sampaio Mei et al. 2013	Only stainless steel (Group A) Stainless steel and elastic ties (Group B)	T0: before installing T1: 30 days after installing T1: 30 days after removal	NA (micron)	
			T0 (A): 66.85 ± 7.70 T1 (A): 52.21 ± 7.33 T2 (A): 61.59 ± 6.76 T0 (B): 64.07 ± 5.80 T1 (B): 53.32 ± 4.54 T2 (B): 59.64 ± 4.17	**T0 (A)** vs. T1 (A), *p* = 0.000122 T1 (A) vs. **T2 (A)**, *p* = 0.000122 **T0 (A)** vs. T2 (A), *p* < 0.05 **T0 (B)** vs. T1 (B), *p* = 0.000122 T1 (B) vs. **T2 (B)**, *p* = 0.000129 **T0 (B)** vs. T2 (B), *p* < 0.05
			CA (micron)	
			T0 (A): 2286.35 ± 227.11 T1 (A): 2654.72 ± 205.88 T2 (A): 2410.22 ± 196.53 T0 (B): 2338.91 ± 188.44 T1 (B): 2608.84 ± 193.38 T2 (B): 2439.37 ± 181.96	T0 (A) vs. **T1 (A)**, *p* = 0.000122 **T1 (A)** vs. T2 (A), *p* = 0.000122 T0 (A) vs. **T2 (A)**, *p* < 0.05 T0 (B) vs. **T1 (B)**, *p* = 0.000122 **T1 (B)** vs. T2 (B), *p* = 0.001028
[40] Arruda et al. 2011	Zones contacting the brackets (Group I) Zones contacting the tubes (Group II) Zones not contacting the appliance (Group III)	T0: 2 years after installing	NA (micron)	
			T0 (I): 52.39 ± 18.86 T0 (II): 53.09 ± 18.32 T0 (III): 63.06 ± 19.46	T0 (I) vs. **T0 (III)**, *p* = 0.000 T0 (II) vs. **T0 (III)**, *p* = 0.000
			CA (micron)	
			T0 (I): 1668.71 ± 578.81 T0 (II): 1884.85 ± 675.03 T0 (III): 1810.66 ± 663.55	T0 (I) vs. **T0 (II)**, *p* = 0.0001 T0 (I) vs. **T0 (III)**, *p* = 0.0001 **T0 (II)** vs. T0 (III), *p* = 0.0001
			Predominant cells (number, %)	
			T0 (I, superficial cells): 11, 50 T0 (II, superficial cells): 19, 86 T0 (III, superficial cells): 10, 45	T0 (II) significant, *p* = 0.0097
[41] Rafighi et al. 2020	—	T0: just after debonding T1: 30 days after T2: 60 days after	NA (micron)	
			T0: 46.62 ± 6.93 T1: 47.51 ± 6.85 T2: 48.57 ± 9.08	T0 vs. **T1**, *p* = 0.000 T0 vs. **T2**, *p* = 0.000 T1 vs. **T2**, *p* = 0.000
			CA (micron)	
			T0: 1747 ± 430 T1: 1767 ± 348 T2: 1847 ± 339	T0 vs. **T1**, *p* = 0.000 T0 vs. **T2**, *p* = 0.000 T1 vs. **T2**, *p* = 0.000
			Predominant cells (number, %)	
			T0 (superficial cells): 22, 71 T0 (intermediate cells): 9, 29	T0 significant, *p* = 0.02
[42] Pereira et al. 2008	Stainless steel (Group A) and ceramic (Group B)	T0: before installing T1: 60 days after placement T2: 30 days after removal	NA (micron)	
			T0: 69.39 ± 19.23 T1 (A): 53.00 ± 17.37 T1 (B): 57.92 ± 19.79 T2 (A): 59.45 ± 20.30 T2 (B): 63.69 ± 20.89	Group A vs. Group A **T0** vs. T1 (A), *p* = 0.00002 **T0** vs. T2 (A), *p* = 0.00003 T1 (A) vs. **T2 (A)**, *p* = 0.00002
				Group B vs. Group B **T0** vs. T1 (B), *p* = 0.00002 T1 (B) vs. **T2 (B)**, *p* = 0.00002
				Group A vs. Group B T1 (A) vs. **T1 (B)**, *p* = 0.00002 T1 (A) vs. **T2 (B)**, *p* = 0.00002 T2 (A) vs. **T2 (B)**, *p* = 0.00002
			CA (micron)	
			T0: 2015.10 ± 696.75 T1 (A): 2473.90 ± 620.03 T1 (B): 2406.10 ± 642.18 T2 (A): 2155.40 ± 677.04 T2 (B): 2041.40 ± 639.44	Group A vs. Group A T0 vs. **T1 (A)**, *p* = 0.00002 T0 vs. **T2 (A)**, *p* = 0.00002 **T1 (A)** vs. T2 (A), *p* = 0.00002
				Group B vs. Group B T0 vs. **T1 (B)**, *p* = 0.00002 T1 (B) vs. **T2 (B)**, *p* = 0.00002
				Group A vs. Group B **T1 (A)** vs. T2 (B), *p* = 0.00002 T2 (A) vs. **T1 (B)**, *p* = 0.00002 **T2 (A)** vs. T2 (B), *p* = 0.00040
			N/C	
			T0: 0.0341 ± 0.01 T1 (A): 0.0224 ± 0.01 T1 (B): 0.0253 ± 0.01 T2 (A): 0.0296 ± 0.01 T2 (B): 0.0336 ± 0.01	Group A vs. Group A **T0** vs. T1 (A), *p* = 0.00002 **T0** vs. T2 (A), *p* = 0.00002 T1 (A) vs. **T2 (A)**, *p* = 0.00002
				Group B vs. Group B **T0** vs. T1 (B), *p* = 0.00002 T1 (B) vs. **T2 (B)**, *p* = 0.00002
				Group A vs. Group B T1 (A) vs. **T1 (B)**, *p* = 0.00002 T1 (A) vs. **T2 (B)**, *p* = 0.00002 **T2 (A)** vs. T1 (B), *p* = 0.00002 T2 (A) vs. **T2 (B)**, *p* = 0.00002
			Predominant cells (number)	
			T0 (superficial cells): 10 T1 (A, superficial cells): 19 T1 (B, superficial cells): 17 T2 (A, superficial cells): 16 T2 (B, superficial cells): 18	T0 vs. **T1 (A, superficial cells)**, *p* < 0.05 T0 vs. **T1 (B, superficial cells)**, *p* < 0.05 T0 vs. **T2 (B, superficial cells)**, *p* < 0.05
[43] Buczko et al. 2017	—	T0: before installing T1: 1 week after installing T2: 24 weeks after installing	NA (micron)	
			T0: 76.36 ± 28.56 T1: 57.89 ± 21.98	**T0** vs. T1, *p* < 0.05
			CA (micron)	
			T0: 1816.77 ± 933.27 T2: 22687.17 ± 617.10	T0 vs. **T2**, *p* < 0.05
			N/C	
			T0: 0.0463 ± 0.015 T1: 0.0361 ± 0.017 T2: 0.0367 ± 0.011	**T0** vs. T1, *p* < 0.05 **T0** vs. T2, *p* < 0.05

Abbreviations: CA, cytoplasmic area; N/C, nuclear/cytoplasmic ratio; NA, nuclear area.

^a^ Bold text indicates that the measurement is greater than the other.

**TABLE 4:** Study variables and significant nuclear alterations observed in the studies included in this review.

Reference	Subgroups	Collection time	Significant values	
			Parameter that showed significant changes	Changes and *p* value^a^
[32] Natarajan et al. 2010	—	T0 (case): after debonding T0 (control): whenever T1 (case): 30 days after debonding T1 (control): 30 days after T0	MN (frequency)	
			T0 (case): 259 ± 233 T0 (controls): 53 ± 51 T1 (case): 48 ± 49	**T0 (case)** vs. T0 (control), *p* < 0.0001 **T0 (case)** vs. T1 (case), *p* < 0.0001
[44] Carrillo‐Novia et al. 2006	—	T0: before placing the appliance T1: 25 days after placement T2: 90 days after placement	MN (frequency)	
			T0: 1.39 ± 1.19 T2: 2.17 ± 1.72	T0 vs. **T2**, *p* = 0.04
			NB (frequency)	
			T0: 4.44 ± 3.77 T2: 7.67 ± 5.97	T0 vs. **T2**, *p* = 0.03
			CC (frequency)	
			T0: 7.72 ± 4.57 T1: 12.89 ± 6.88 T2: 14.78 ± 8.47	T0 vs. **T1**, *p* = 0.01 T0 vs. **T2**, *p* = 0.00
			KL (frequency)	
			T1: 1.56 ± 1.38 T2: 2.89 ± 2.98	T1 vs. **T2**, *p* = 0.05
			CC + KL + KR + PN (frequency)	
			T0: 11.33 ± 6.40 T1: 16.44 ± 7.96 T2: 20.61 ± 10.7 T0 (female): 13.8 ± 7.8 T0 (male): 21.6 ± 5.4	T0 vs. **T1**, *p* = 0.05 T1 vs. **T2**, *p* = 0.01 T0 (female) vs. **T0 (male)**, *p* = 0.03
[45] Francis et al. 2017	—	T0: before the installation T1: 7 days after installation T2: 30 days after installation T3: 45 days after installation	MN (frequency)	
			T0: 1.2 ± 0.25 T1: 3.4 ± 0.37 T2: 2.4 ± 0.24 T3: 2.1 ± 0.11	T0 vs. **T1**, *p* < 0.001 T0 vs. **T2**, *p* < 0.001 T0 vs. **T3**, *p* < 0.001 T0 vs. **T4**, *p* < 0.001
[46] Cunha et al. 2018	—	T0: before placing the appliance T1: 1 month after placement T2: 3 months after placement	Normal cell (frequency)	
			T0: 961.40 ± 35.05 T1: 959.40 ± 9.10 T2: 930.00 ± 13.14	**T0** vs. T2, *p* < 0.0001
			PK (frequency)	
			T0: 1.17 ± 1.13 T1: 3.50 ± 2.18 T2: 6.97 ± 4.52	T0 vs. **T1**, *p* < 0.01 T0 vs. **T2**, *p* < 0.01 T1 vs. **T2**, *p* < 0.01
			KL (frequency)	
			T0: 11.46 ± 3.09 T1: 16.39 ± 5.72 T2: 27.46 ± 8.05	T0 vs. **T1**, *p* < 0.01 T0 vs. **T2**, *p* < 0.01 T1 vs. **T2**, *p* < 0.01
			BN (frequency)	
			T0: 10.07 ± 3.72 T1: 15.39 ± 4.81 T2: 31.89 ± 7.01	T0 vs. **T1**, *p* < 0.01 T0 vs. **T2**, *p* < 0.01 T1 vs. **T2**, *p* < 0.01
			NB (frequency)	
			T0: 0.71 ± 1.21 T1: 3.50 ± 2.23 T2: 1.71 ± 1.21	T0 vs. **T1**, *p* < 0.01 **T1** vs. T2, *p* < 0.01
[47] Gonçalves et al. 2015	—	*Buccal comet assay* T0: 1 week before installation T1: 14 days after installation *Micronucleus cytome assay* T0: day of installation T1: 28 days after installation T2: 6 months after installation T3: 1 year after installation	None	
			—	—
[48] Angelieri et al. 2011	—	T0: before the installation T1: during the therapy (around 170 days after the installation) T2: after the therapy (at least after 6 months)	None	
			—	—
[49] Flores‐Bracho et al. 2019	—	T0 (control): whenever T0 (I): during the 1–12 months long treatment T0 (II): during the 13–24 months long treatment T0 (III): during the 25–48 months long treatment T0 (IV): during the more than 48 months long treatment	KL (median, confidence limits for free distribution—95%)	
			T0 (*control*): 5, 2–10 T0 (*I*): 4, 0–9 T0 (*II*): 2, 0–4 T0 (*III*): 2, 0–5 T0 (*IV*) 0, 0–4	T0 (*control*) vs. **T0 (*I*)**, *p* = 0.0166 T0 (*control*) vs. **T0 (*II*)**, *p* = 0.0166 T0 (*control*) vs. **T0 (*III*)**, *p* = 0.0166 **T0 (*control*)** vs. **T0 (*IV*)**, *p* = 0.0166
[50] Westphalen et al. 2008	—	T0: before the installation T1: 30 days after installation	Number of cases with MN (%)	
			T0: 0 T1: 25	T0 vs. **T1**, *p* = 0.0213

Abbreviations: CC, condensed chromatin; KL, karyolysis; KR, karyorrhexis; NB, nuclear buds; PN, pyknosis.

^a^ Bold text indicates that the measurement is greater than the other.

**TABLE 5:** Study variables and significant cytotoxic and genotoxic alterations observed in the studies included in this review.

Reference	Subgroups	Collection time	Significant values	
			Parameter that showed significant changes	Changes and *p* value^a^
[33] Hafez et al. 2011	Stainless steel (Group I) Stainless steel and Ni‐Ti alloy (Group II) Stainless steel and titanium (Group III) Ni‐Ti alloy and titanium (Group IV) Nothing (control)	Inner part of buccal mucosa T0: Before the installation T1: 3 months after installation T2: 6 months after installation	Viability (%) Case vs. case	
			T0 (mean): 8.11 ± 6.14 T2 (mean): 4.51 ± 2.77 T0 (I): 10.2 ± 8.0 T1 (I): 7.8 ± 6.4 T2 (I): 4.1 ± 2.9 T0 (IV): 9.0 ± 2.3 T1 (IV): 6.3 ± 2.1 T2 (IV): 4.1 ± 3.1	**T0 (mean)** vs. T2 (case), *p* = 0.009 **T0 (I)** vs. T1 (I), *p* = 0.035 **T0 (I)** vs. T2 (I), *p* = 0.035 **T0 (IV)** vs. T1 (IV), *p* = 0.033 **T0 (IV)** vs. T2 (IV), *p* = 0.011
			Composite score Case vs. case	
			T0 (mean): 125.6 ± 46.5 T2 (mean): 98.8 ± 33.7 T0 (II): 124 ± 33.4 T1 (II): 68 ± 18.1 T2 (II): 92 ± 8.0 T0 (IV): 142.3 ± 63.5 T2 (IV): 90.3 ± 24.0	**T0 (mean)** vs. T2 (mean), *p* = 0.009 **T0 (II)** vs. T1 (II), *p* = 0.016 **T0 (II)** vs. T2 (II), *p* = 0.043 **T0 (IV)** vs. T2 (IV), *p* = 0.017
			Composite score Control vs. control	
			T0 (control): 108 ± 30.9 T1 (control): 50.9 ± 27.1	**T0 (control)** vs. T1 (control), *p* = 0.000
			Damage frequency (%) Case vs. case	
			T0 (III): 38.7 ± 2.3 T1 (III): 48.0 ± 2.7 T2 (III): 56.3 ± 6.8 T0 (IV): 57.0 ± 12.7 T1 (IV): 46.3 ± 20.5 T2 (IV): 36.5 ± 7.9	T0 (III) vs. **T1 (III)**, *p* = 0.000 T0 (III) vs. **T2 (III)**, *p* = 0.005 **T0 (IV)** vs. T1 (IV), *p* = 0.038 **T0 (IV)** vs. T2 (IV), *p* = 0.003
			Damage frequency (%) Control vs. control	
			T0 (control): 37.3 ± 11.1 T1 (control): 27.4 ± 10.4	**T0 (control)** vs. T1 (control), *p* = 0.003
[34] Kapadia et al. 2018	—	Buccal mucosa T0: before installation T1: 5 months after installation T2: 10 months after installation T3: 15 months after installation T4: 20 months after installation	None	
			—	**—**
			Head diameter (px)	
			T0: 110.5 T1: 139.4 T2: 156.8 T3: 163.1 T4: 180.2	T0 vs. **all**, *p* = 0.000
			DNA in tail (%)	
			T0: 14.02 T1: 16.88 T2: 21.08 T3: 28.41 T4: 30.82	T0 vs. **all**, *p* = 0.001
			Tail lenght (%)	
			T0: 14.87 T1: 20.49 T2: 24.34 T3: 30.21 T4: 35.60	T0 vs. **all**, *p* = 0.002
[35] Faccioni et al. 2003	Stainless steel, Ni‐Ti alloy or titanium (case) None (control)	Inner part of cheek mucosa T0 (case): from 2 to 4 years after the installation T0 (control): whenever	Viability (%)	
			T0 (control): 73.43 ± 12.29 T0 (case): 50.40 ± 13.55	T0 (control): 73.43 ± 12.29 T0 (case): 50.40 ± 13.55
			Tail length (micron)	
			T0 (control): 10.54 ± 2.41 T0 (case): 15.56 ± 6.78	T0 (control): 10.54 ± 2.41 T0 (case): 15.56 ± 6.78
			Tail moment (px)	
			T0 (control): 0.30 ± 0.09 T0 (case): 0.46 ± 0.21	T0 (control): 0.30 ± 0.09 T0 (case): 0.46 ± 0.21
			Damage frequency (%)	
			T0 (control): 11.43 ± 6.58 T0 (case): 17.62 ± 10.08	T0 (control): 11.43 ± 6.58 T0 (case): 17.62 ± 10.08
[37] Fernández‐Miñano et al. 2011	Stainless steel (Group I) Titanium (Group II) Nickel‐free metal (Group III)	Inner part of cheek mucosa T0: before installation T1: 30 days after installation	Tail moment (px)	
			T0: 25.87 ± 3.41 T1 (I): 69.35 11.68 T1 (III): 68.41 ± 26.63	T0 vs. **T1 (I)**, *p* < 0.001 T0 vs. **T1 (III)**, *p* < 0.001
[47] Gonçalves et al. 2015	—	Buccal mucosa *Buccal comet assay* T0: 1 week before installation T1: 14 days after installation *Micronucleus cytome assay* T0: day of installation T1: 28 days after installation T2: 6 months after installation T3: 1 year after installation	Damage frequency (%)	
			T0: 35.94 ± 15.42 T1: 53.25 ± 19.40	T0 vs. **T1**, *p* = 0.0071
			Damage index	
			T0: 50.31 ± 25.47 T1: 75.69 ± 37.69	T0 vs. **T1**, *p* = 0.0280
[50] Westphalen et al. 2008	—	Mucosa of lower lip T0: before the installation T1: 30 days after installation	None	
			—	**—**

^a^ Bold text indicates that the measurement is greater than the other.

Table S7 presents the inclusion and exclusion criteria of the studies included in this systematic review. The most common exclusion criteria for the patients assessed in the included studies were the presence of systemic diseases, smoking, alcohol consumption, metal allergies, presence of dental restorations, and previous orthodontic treatments. Indeed, the presence of one or more of these features could alter the cytological analysis by increasing inflammation and/or decreasing cellular vitality. Table S8 describes the procedure for sample collection, treatment, and assessment of the studies included in this review. Table S9 groups all the studies that used light microscopy and BCA analysis and reports all the staining techniques that have been used, as well as the number of cells that have been analyzed. A detailed overview of the evidence obtained from the studies included in this review is presented in Table S10. Table S11 summarizes all the variables assessed in the studies included in this review and the statistical analysis used.

## 4 Discussion

Different studies have assessed cellular alterations following the presence of a fixed OA, including metal cellular content, cytomorphological and cytomorphometric alterations, nuclear alterations, and DNA damage.

### 4.1 Cellular Metal Content

OAs are in continuous contact with the oral mucosa, leading to the gradual release of metal ions. Localized corrosion can be attributable to plaque, tartar, microorganisms' acidic products, food, drinks, inflated levels of oxygen and chloride in the saliva, forces acting on the OA, and friction between mucosa, brackets, and arches [33, 35, 37]. The maximum release of metal ions from OA has been observed to be higher in the first 4 or 5 months after the installation, with a consequent systemic distribution. Their levels then decreased because of the formation of a metal‐oxide film around the components of the appliance [32, 35, 36, 37].

FOT causes an increase in nickel cellular content, even after the peak. Despite the prolonged presence of the metal in the cells, toxic limit levels were never reached, neither at the peak period nor after [33, 34, 35, 36]. Nevertheless, the relative risk (RR) analysis showed no significant differences between the experimental and control groups, indicating cellular adaptation or post‐damage repair processes [33]. Higher concentrations of chromium have not been found as commonly as for nickel, but some authors reported significant increases after 30 days, 3 months, and 6 months, despite depending on the OA composition [33, 37]. The RR analysis shows a significant difference in chromium concentration between the control group and the experimental group at 3 months [33]. Cobalt, titanium, manganese, and iron have been assessed by only one study each. Despite showing a significant increase in the cellular concentration when compared to a control group, these changes were dependent on the OA composition [35, 37]. No significant differences in metal content after debonding have been seen for nickel [32] or chromium [32, 36]. No studies have assessed cobalt, titanium, manganese, and iron levels after debonding.

Nickel‐titanium and nickel‐free metal, mostly stainless steel, are the materials that showed a constant and higher release when different compositions were compared [33, 37].

### 4.2 Cytological Changes

Hyperplasia and hyperkeratinization are part of the adaptive response of the oral mucosa cells to the presence of an appliance [39, 40]. The mechanical stress due to the OA can lead to a diminished blood supply and inadequate nutrition or hypoxia, resulting in the changes [40].

Among the studies considered, none of them showed an increase in the N/C ratio of squamous cells. Alsahi et al. found a decrease in cytoplasmic area (CA) within the cells of the patients, but it was also accompanied by a diminution in nuclear area (NA), consistent with atrophy rather than dysplastic changes [38]. NA showed more commonly a decrease after positioning, while CA tended to increase after the OA was installed [38, 39, 40, 42, 43], probably due to an augmentation of cell metabolism, which is necessary for the adaptive response [39]. Also, keratinized cells have a smaller nucleus and a larger cytoplasm, a feature that justifies these data. The N/C ratio showed a diminution in all the studies considered, but this finding was significant only in two of them [42, 43]. After the OA was removed, these changes showed a significant tendency to regress, even if without a comeback to the original value, therefore supporting the reversibility of cytomorphological changes [39, 41, 42].

An increase in the number of superficial cells in the smears after positioning of the OA has been reported, with a predominance of sub‐superficial cells because of hyperplasia and hyperkeratinization [39, 40, 41, 42]. When the OA was removed, this variation regressed significantly, even if it did not reach the original levels before the positioning [39, 41]. None of the smears analyzed in the studies considered here had basal or para‐basal cells [39, 40, 41, 42]. This finding is not surprising since the number of basal or para‐basal cells is normally low in the exfoliative cytology, due to the superficial nature of this sampling technique [40, 41].

In subjects undergoing FOT, no Pap Classes III, IV, or V have been reported, but rather a prevalence of Classes I and II, with no suspicious alterations [39, 42]. The presence of Pap Class II in the oral smears must be considered physiological, as the inflammatory response to OA is part of the normal adaptation process to it and may cause morphologic atypia; hygiene reinforcements and a good oral hygiene routine could reduce the inflammation [38, 40]. Papanicolaou has been the most used staining technique for cytological changes assessment, as well as for nuclear alteration changes [32, 34, 38, 39, 40, 41, 42, 44, 45], followed by Feulgen/Fast green [46, 47, 48, 49] and by May‐Grunwald‐Giemsa [43, 50]. The authors have analyzed a number of cells per patient that varied between 50 [39, 42, 43], 1000 [32, 44, 46, 47, 48, 50], and 2000 [48, 49]. Some authors did not specify how many cells per patient have been analyzed [34, 38, 40, 41, 45]. These differences between the stainings that have been used could influence the results, as well as a bigger number of analyzed cells could reduce false‐positive or false‐negative results.

### 4.3 Nuclear Alterations

The MN assay is an optimal way to assess genetic damage because of its minimal invasiveness, ease of scoring, limited costs, limited time required, and precision [32, 48]. In addition, the MN assay is way more sensitive than BCA at detecting DNA damage; buccal mucosa cells have limited DNA repair capacities, making them an accurate sample for early genomic instability [48, 50]. DNA‐specific stainings (such as Feulgen/Fast green) are recommended to reduce false‐positive results [46, 47]. Different factors can influence MN assay results, such as age, smoking, and drinking [48, 50].

A significant increase in MN frequency has been observed after a few days [45], a month [50], and 3 months from OA placement [44]. In addition, Novia et al. pointed out a lower MN frequency in the control group when compared to smears collected 30 days after the OA was removed. This confirms the persistence of genetic damage even at the end of the orthodontic treatment [32]; nonetheless, the longer‐term assessment showed a decrease in MN frequency, supporting the reversibility of the process [32, 48]. However, other authors found no significance in MN increased frequency as well as in decrease after debonding between case and control groups [46, 47, 49]. The absence of a steadily consistent correlation between OA and genetic alterations could be due to the good efficiency and adaptability of the patient's DNA repair mechanisms, metal release, and induced damages that are not clinically sufficient to cause damage to the patient [49].

The presence of nuclear buds (NB) can also indicate DNA damage. A significant increase in NB frequency after the OA installation was reported by some authors [44, 46], providing evidence of genome damage caused by mispaired DNA breaks or telomere fusion. No studies about the regression of NB frequency after debonding are available.

Other authors assessed DNA damage indicators of cellular death [44, 46, 47, 48, 49], including condensed chromatin (CC), karyorrhexis (KR), karyolitic (KL), and pyknosis (PK), and all can be assessed together. The CC increase during FOT was significant only in one study, while no significant difference in KR frequency was found. KL frequency increased at all time points after OA installation in different studies [44, 46]. When a control group was compared with subjects undergoing long‐term FOT (e.g., more than 48 months), the first had a higher KL frequency, suggesting that the adaptation process enables reaching levels comparable to those before treatment [49]. Two studies assessed CC, KR, KL, and PK together as a single parameter, encountering an increase, though it was significant only in one case [44]. Cellular death indicator frequency seems like a worse parameter than MN frequency to identify genetic damage, due to its inconstancy in variations.

### 4.4 Cytotoxic Effects

Longitudinal data based on the trypan blue dye test suggest a decrease in cell viability after the installation of OA [33, 34]. Nevertheless, Hafez et al.'s results did not show significance, likely because of the basal low vitality in superficial normal buccal mucosa cells (due to mechanical stress) [34]; this is one of the limitations of the exfoliative and superficial cytology. A case–control study by Faccioni et al. [35] found significantly higher viability in the control group. The Mann–Whitney *U*‐test showed that the decrease in cellular viability had a significant inverse correlation with nickel and cobalt levels, underscoring how the presence of the OA in the oral cavity releases metal ions sufficient to induce cytotoxic effects [35]. None of the studies found a significant increase in cell vitality after an initial significant decrease, nor did they assess the cell vitality after the removal of the appliance. Lastly, none of the authors executing a trypan blue vitality test stated how many cells per patient had been assessed [33, 34, 35].

### 4.5 Genotoxic Effects

BCA consists of an electrophoresis of the nucleoids extracted from the sampled cells that measures DNA damage at a cellular level. DNA damage can take the form of DNA fragmentation (with greater comet migration) or DNA–DNA and DNA–protein crosslinking (with lower migration).

Damage frequency (DF), defined as the number of comets per 100 nucleoids examined, has shown an increase after OA installation [33, 35, 47]. Titanium OAs are the only ones showing a significant decrease in DF at all time points, confirming their higher biocompatibility. Hafez et al. have reported an inverse correlation between cellular metal content and a positive correlation between metal content and damage frequency. The RR analysis showed a significant difference between the experimental group when compared with the control group, but only after 3 months. These analyses suggest that the presence of an OA induces DNA damage but that the composition of the OA is not as relevant [33].

Given a number (“*x*”) of nucleoids, the damage index (DI) is defined as the sum of the grade of each nucleoid, multiplied by “*x*.” DI was assessed in two studies [47, 50]: the results showed an increase in DI after OA installation, which was significant only in one study [47].

Hafez et al. also measured the composite score, obtained as a combination of DF and DI (composite score, CS), which gives an overview of the genotoxicity of a substance. The average score of the test group was significantly lower after 6 months, supposedly indicating low DNA damage. The authors underlined that, since the decrease in the composite score was not accompanied by a decrease in DF, the damage likely took the form of DNA crosslinking. The RR analysis indicated a significant difference in the changes between the case and the control group, but only after 3 months, suggesting a subsequent cellular adaptation or repair process. The linear regression analysis did not find any relationship between the metal level and the cytotoxic or genotoxic effects in the sample [33]. No data about DF, DI, and composite score variations after the OA removal were available.

Morphometric DNA damage parameters tend to increase significantly after the installation of an OA, specifically tail length [34, 35], DNA in the tail [34], and tail moment [35, 37]. In one analysis, tail length was significantly correlated with cobalt cellular concentration [35]. Apart from the increase in head diameter observed in one case [34], most of the results indicate the presence of DNA damage. Differently from the parameters obtained via visual assessment (e.g., DF, DI, and CS), morphometric parameters tend to show a more constant degree of DNA damage [34, 35, 37]. Among the studies considering comet morphometric data, none of them assessed the parameters after the removal of the OA, nor did they find a tendency to reversibility at different time points.

Among the BCA analysis, the most used staining was ethidium bromide [33, 35, 37], followed by silver nitrate [47, 50]. One of the included studies did not report the staining that has been used for microscopy observation [34]. The authors assessed a number of cells per patient among 50 [33, 50], 100 [35, 47] and 200 [37]. One study did not report this information [34].

### 4.6 Limitations and Future Research

Current data suggest low damage levels without dysplastic changes following an OA implantation. Nevertheless, future research should be addressed to investigate the long‐term evolution and reversibility of the cytotoxic and genotoxic changes. Data about the influence of OA composition and structural design on the resulting damage to the oral mucosa are still lacking and need to be investigated.

## 5 Conclusion

Cellular alterations in the oral cavity can be analyzed using different methods, ranging from light microscopy to automated electrophoresis systems. Different studies confirmed the presence of increased metal ion levels in the smears after the placement of the OA, with higher release in the earliest months. Cytological changes have been observed, as well as nuclear abnormalities, but a reversible change trend has been detected after the removal of the fixed OA. BCA, an instrument assessing genotoxic effects, showed increased DNA damage in the patients wearing the OA, even if not constantly, among the studies. Some of the analyses performed in the included studies revealed nonsignificant cellular changes or did not find any changes at all. This could be a result of the adaptability of epithelial cells, the low cellular toxicity of OAs, or a combination of both. Importance should be given to the staining technique that has been used and to the number of cells per patient that have been analyzed. Nevertheless, considering the existence of significant changes among the literature, progress in OA materials and design will help reduce the risk of cellular and genetic alterations, empowering orthodontic treatment safety.

## Author Contributions

**Francesco Paolo Modugno, Letterio Runza, and Matteo Pellegrini:** software, supervision, visualization, writing – original draft, and writing – review and editing. **Elisabetta Kuhn:** conceptualization, data curation, formal analysis, investigation, methodology, project administration, resources, validation, and writing – review and editing. **Chiara Luisa Bianchi:** conceptualization, data curation, formal analysis, investigation, methodology, project administration, and resources. **Federica Pulicari:** software, supervision, visualization, and writing – review and editing. **Francesco Spadari:** conceptualization, data curation, formal analysis, investigation, methodology, project administration, resources, supervision, validation, and writing – review and editing. All authors commented on previous versions of the manuscript. All authors have read and agreed to the published version of the manuscript.

## Conflicts of Interest

The authors declare no conflicts of interest.

## Peer Review

The peer review history for this article is available at https://www.webofscience.com/api/gateway/wos/peer‐review/10.1111/jop.13643.

## Supporting information


**Table S1.** Search strategies for electronic databases.
**Table S2.** PRISMA 2020 Checklist.
**Table S3.** Summary table of studies excluded in this systematic review.
**Table S4.** Bias domains included in the ROBINS‐I tool.
**Table S5.** Bias analysis of the studies included in this review using the ROBINS‐I tool for Observational Studies.
**Table S6.** NHLBI Quality Assessment Tool for Observational Cohort Studies.
**Table S7.** Baseline characteristics of the patients, study design inclusion criteria, and exclusion criteria of the studies considered in this review.
**Table S8.** Sample collection, treatment, and assessment of the studies included in this review.
**Table S9.** Staining techniques and number of cells assessed per patient in cytological and nuclear assessment, and for BCA.
**Table S10.** Evidence of studies included in this systematic review.
**Table S11.** Parameters assessed and statistical analysis performed in the studies included in this review.

## Data Availability

The data that support the findings of this study are available from the corresponding author upon reasonable request.
