# Supplementary material for: Biological Consequences and Assessment Methods Analysis of Fixed Orthodontic Appliances on Oral Epithelial Cells: A Systematic Review
Source: J Oral Pathol Med. 2025 May 19;54(6):413–34. doi: 10.1111/jop.13643 (PMC12230921; doi:10.1111/jop.13643)
Supplement: Supplementary file 1 — Table S1. Search strategies for electronic databases. Table S2. PRISMA 2020 Checklist. Table S3. Summary table of studies excluded in this systematic review. Table S4. Bias domains included in the ROBINS‐I tool. Table S5. Bias analysis of the studies included in this review using the ROBINS‐I tool for Observational Studies. Table S6. NHLBI Quality Assessment Tool for Observational Cohort Studies. Table S7. Baseline characteristics of the patients, study design inclusion criteria, and exclusion criteria of the studies considered in this review. Table S8. Sample collection, treatment, and assessment of the studies included in this review. Table S9. Staining techniques and number of cells assessed per patient in cytological and nuclear assessment, and for BCA. Table S10. Evidence of studies included in this systematic review. Table S11. Parameters assessed and statistical analysis performed in the studies included in this review. [file JOP-54-413-s001.docx]

**Table S1.** PRISMA 2020 Checklist

| **Section and Topic** | **Item #** | **Checklist item** | **Location where item is reported** |
| --- | --- | --- | --- |
| **TITLE** | | |  |
| Title | 1 | Identify the report as a systematic review. | Title |
| **ABSTRACT** | | |  |
| Abstract | 2 | See the PRISMA 2020 for Abstracts checklist. |  |
| **INTRODUCTION** | | |  |
| Rationale | 3 | Describe the rationale for the review in the context of existing knowledge. | Initial introduction |
| Objectives | 4 | Provide an explicit statement of the objective(s) or question(s) the review addresses. | End of introduction |
| **METHODS** | | |  |
| Eligibility criteria | 5 | Specify the inclusion and exclusion criteria for the review and how studies were grouped for the syntheses. | Dedicated section in  M&M |
| Information sources | 6 | Specify all databases, registers, websites, organisations, reference lists and other sources searched or consulted to identify studies. Specify the date when each source was last searched or consulted. | Dedicated section in  M&M |
| Search strategy | 7 | Present the full search strategies for all databases, registers and websites, including any filters and limits used. | Dedicated section in  M&M |
| Selection process | 8 | Specify the methods used to decide whether a study met the inclusion criteria of the review, including how many reviewers screened each record and each report retrieved, whether they worked independently, and if applicable, details of automation tools used in the process. | Dedicated section in  M&M |
| Data collection process | 9 | Specify the methods used to collect data from reports, including how many reviewers collected data from each report, whether they worked independently, any processes for obtaining or confirming data from study investigators, and if applicable, details of automation tools used in the process. | Dedicated section in  M&M |
| Data items | 10a | List and define all outcomes for which data were sought. Specify whether all results that were compatible with each outcome domain in each study were sought (e.g. for all measures, time points, analyses), and if not, the methods used to decide which results to collect. | Dedicated section in  M&M |
|  | 10b | List and define all other variables for which data were sought (e.g. participant and intervention characteristics, funding sources). Describe any assumptions made about any missing or unclear information. | Dedicated section in  M&M |
| Study risk of bias assessment | 11 | Specify the methods used to assess risk of bias in the included studies, including details of the tool(s) used, how many reviewers assessed each study and whether they worked independently, and if applicable, details of automation tools used in the process. | 2 reviewers assessed  the risk of bias – specified in M&M |
| Effect measures | 12 | Specify for each outcome the effect measure(s) (e.g. risk ratio, mean difference) used in the synthesis or presentation of results. | Mean difference (M&M) |
| Synthesis methods | 13a | Describe the processes used to decide which studies were eligible for each synthesis (e.g. tabulating the study intervention characteristics and comparing against the planned groups for each synthesis (item #5)). | Type of intervention |
|  | 13b | Describe any methods required to prepare the data for presentation or synthesis, such as handling of missing summary statistics, or data conversions. | Procedure described M&M |
|  | 13c | Describe any methods used to tabulate or visually display results of individual studies and syntheses. | Procedure described M&M |
|  | 13d | Describe any methods used to synthesize results and provide a rationale for the choice(s). If meta-analysis was performed, describe the model(s), method(s) to identify the presence and extent of statistical heterogeneity, and software package(s) used. | Answer to PICO |
|  | 13e | Describe any methods used to explore possible causes of heterogeneity among study results (e.g. subgroup analysis, meta-regression). | N/A |
|  | 13f | Describe any sensitivity analyses conducted to assess robustness of the synthesized results. | N/A |
| Reporting bias assessment | 14 | Describe any methods used to assess risk of bias due to missing results in a synthesis (arising from reporting biases). | N/A |
| Certainty assessment | 15 | Describe any methods used to assess certainty (or confidence) in the body of evidence for an outcome. | Reported conclusions of the included studies |
| **RESULTS** | | |  |
| Study selection | 16a | Describe the results of the search and selection process, from the number of records identified in the search to the number of studies included in the review, ideally using a flow diagram. | Dedicated table |
|  | 16b | Cite studies that might appear to meet the inclusion criteria, but which were excluded, and explain why they were excluded. | Dedicated table |
| Study characteristics | 17 | Cite each included study and present its characteristics. | Dedicated table |
| Risk of bias in studies | 18 | Present assessments of risk of bias for each included study. | Dedicated table |
| Results of individual studies | 19 | For all outcomes, present, for each study: (a) summary statistics for each group (where appropriate) and (b) an effect estimate and its precision (e.g. confidence/credible interval), ideally using structured tables or plots. | Dedicated table |
| Results of syntheses | 20a | For each synthesis, briefly summarise the characteristics and risk of bias among contributing studies. | Dedicated table |
|  | 20b | Present results of all statistical syntheses conducted. If meta-analysis was done, present for each the summary estimate and its precision (e.g. confidence/credible interval) and measures of statistical heterogeneity. If comparing groups, describe the direction of the effect. | N/A |
|  | 20c | Present results of all investigations of possible causes of heterogeneity among study results. | Dedicated table |
|  | 20d | Present results of all sensitivity analyses conducted to assess the robustness of the synthesized results. | Dedicated table |
| Reporting biases | 21 | Present assessments of risk of bias due to missing results (arising from reporting biases) for each synthesis assessed. | Dedicated table |
| Certainty of evidence | 22 | Present assessments of certainty (or confidence) in the body of evidence for each outcome assessed. | Dedicated table |
| **DISCUSSION** | | |  |
| Discussion | 23a | Provide a general interpretation of the results in the context of other evidence. | Followed |
|  | 23b | Discuss any limitations of the evidence included in the review. | Followed |
|  | 23c | Discuss any limitations of the review processes used. | Followed |
|  | 23d | Discuss implications of the results for practice, policy, and future research. | Followed |
| **OTHER INFORMATION** | | |  |
| Registration and protocol | 24a | Provide registration information for the review, including register name and registration number, or state that the review was not registered. | DOI No 10.17605/OSF.IO/25MZ7 |
|  | 24b | Indicate where the review protocol can be accessed, or state that a protocol was not prepared. | OSF |
|  | 24c | Describe and explain any amendments to information provided at registration or in the protocol. | N/A |
| Support | 25 | Describe sources of financial or non-financial support for the review, and the role of the funders or sponsors in the review. | None |
| Competing interests | 26 | Declare any competing interests of review authors. | None |
| Availability of data, code and other materials | 27 | Report which of the following are publicly available and where they can be found: template data collection forms; data extracted from included studies; data used for all analyses; analytic code; any other materials used in the review. | N/A |

**Table S2.** Search Strategies for electronic databases.

| **Database** | **Search strategy** |
| --- | --- |
| PubMed (MEDLINE) | #1 “Cytology” [MESH] OR (Cytopathology) OR (Cytopathologies)  #2 “Epithelial Cells” [MESH] OR (Cell, Epithelial) OR (Cells, Epithelial) OR (Epithelial Cell) OR (Squamous Epithelial Cells) OR (Cell, Squamous Epithelial) OR (Cells, Squamous Epithelial) OR (Epithelial Cell, Squamous) OR (Epithelial Cells, Squamous) OR (Squamous Epithelial Cell) OR (Squamous Cells) OR (Cell, Squamous) OR (Cells, Squamous) OR (Squamous Cell)  #3 “Mouth Mucosa” [MESH] OR (Mucosa, Mouth) OR (Oral Mucosa) OR (Mucosa, Oral) OR (Buccal Mucosa)  #4 “DNA Damage” [MESH] OR (DNA Lesions) OR (DNA Lesion) OR (Injury, DNA) OR (DNA Injuries) OR (DNA Injury) OR (Genotoxic Stress) OR (Stress, Genotoxic)  #5 “Mutagens” [MESH] OR (Genotoxins) OR (Mutagen) OR (Genotoxin) OR (Clastogens) OR (Clastogen)  #6 “Cytotoxins” [MESH] OR (Cytotoxin) OR (Cytotoxic Agents) OR (Cytotoxic Agent) OR (Agent, Cytotoxic) OR (Cytolysins)  #7 “Mutagenicity Tests” [MESH] OR (Mutagenicity Test) OR (Genetic Toxicity Tests) OR (Genotoxicity Tests) OR (Genotoxicity Test) OR (Test, Genotoxicity) OR (Tests, Genotoxicity) OR (Mutagen Screening) OR (Mutagen Screenings) OR (Screening, Mutagen) OR (Screenings, Mutagen) OR (Tests, Genetic Toxicity) OR (Genetic Toxicity Test) OR (Toxicity Test, Genetic) OR (Toxicity Tests, Genetic)  #8 “Orthodontic Appliances” [MESH] OR “Appliance, Orthodontic” OR “Appliances, Orthodontic” OR “Orthodontic appliance”  #9 (#1 OR #2 OR #3) AND #4 AND #8  #10 (#1 OR #2 OR #3) AND #5 AND #8  #11 (#1 OR #2 OR #3) AND #6 AND #8  #12 (#1 OR #2 OR #3) AND #7 AND #8 |
| SCOPUS | #1 “Cytology” [MESH] OR (Cytopathology) OR (Cytopathologies)  #2 “Epithelial Cells” [MESH] OR (Cell, Epithelial) OR (Cells, Epithelial) OR (Epithelial Cell) OR (Squamous Epithelial Cells) OR (Cell, Squamous Epithelial) OR (Cells, Squamous Epithelial) OR (Epithelial Cell, Squamous) OR (Epithelial Cells, Squamous) OR (Squamous Epithelial Cell) OR (Squamous Cells) OR (Cell, Squamous) OR (Cells, Squamous) OR (Squamous Cell)  #3 “Mouth Mucosa” [MESH] OR (Mucosa, Mouth) OR (Oral Mucosa) OR (Mucosa, Oral) OR (Buccal Mucosa)  #4 “DNA Damage” [MESH] OR (DNA Lesions) OR (DNA Lesion) OR (Injury, DNA) OR (DNA Injuries) OR (DNA Injury) OR (Genotoxic Stress) OR (Stress, Genotoxic)  #5 “Mutagens” [MESH] OR (Genotoxins) OR (Mutagen) OR (Genotoxin) OR (Clastogens) OR (Clastogen)  #6 “Cytotoxins” [MESH] OR (Cytotoxin) OR (Cytotoxic Agents) OR (Cytotoxic Agent) OR (Agent, Cytotoxic) OR (Cytolysins)  #7 “Mutagenicity Tests” [MESH] OR (Mutagenicity Test) OR (Genetic Toxicity Tests) OR (Genotoxicity Tests) OR (Genotoxicity Test) OR (Test, Genotoxicity) OR (Tests, Genotoxicity) OR (Mutagen Screening) OR (Mutagen Screenings) OR (Screening, Mutagen) OR (Screenings, Mutagen) OR (Tests, Genetic Toxicity) OR (Genetic Toxicity Test) OR (Toxicity Test, Genetic) OR (Toxicity Tests, Genetic)  #8 “Orthodontic Appliances” [MESH] OR “Appliance, Orthodontic” OR “Appliances, Orthodontic” OR “Orthodontic appliance”  #9 (#1 OR #2 OR #3) AND #4 AND #8  #10 (#1 OR #2 OR #3) AND #5 AND #8  #11 (#1 OR #2 OR #3) AND #6 AND #8  #12 (#1 OR #2 OR #3) AND #7 AND #8 |
| Web of Science | #1 “Cytology” [MESH] OR (Cytopathology) OR (Cytopathologies)  #2 “Epithelial Cells” [MESH] OR (Cell, Epithelial) OR (Cells, Epithelial) OR (Epithelial Cell) OR (Squamous Epithelial Cells) OR (Cell, Squamous Epithelial) OR (Cells, Squamous Epithelial) OR (Epithelial Cell, Squamous) OR (Epithelial Cells, Squamous) OR (Squamous Epithelial Cell) OR (Squamous Cells) OR (Cell, Squamous) OR (Cells, Squamous) OR (Squamous Cell)  #3 “Mouth Mucosa” [MESH] OR (Mucosa, Mouth) OR (Oral Mucosa) OR (Mucosa, Oral) OR (Buccal Mucosa)  #4 “DNA Damage” [MESH] OR (DNA Lesions) OR (DNA Lesion) OR (Injury, DNA) OR (DNA Injuries) OR (DNA Injury) OR (Genotoxic Stress) OR (Stress, Genotoxic)  #5 “Mutagens” [MESH] OR (Genotoxins) OR (Mutagen) OR (Genotoxin) OR (Clastogens) OR (Clastogen)  #6 “Cytotoxins” [MESH] OR (Cytotoxin) OR (Cytotoxic Agents) OR (Cytotoxic Agent) OR (Agent, Cytotoxic) OR (Cytolysins)  #7 “Mutagenicity Tests” [MESH] OR (Mutagenicity Test) OR (Genetic Toxicity Tests) OR (Genotoxicity Tests) OR (Genotoxicity Test) OR (Test, Genotoxicity) OR (Tests, Genotoxicity) OR (Mutagen Screening) OR (Mutagen Screenings) OR (Screening, Mutagen) OR (Screenings, Mutagen) OR (Tests, Genetic Toxicity) OR (Genetic Toxicity Test) OR (Toxicity Test, Genetic) OR (Toxicity Tests, Genetic)  #8 “Orthodontic Appliances” [MESH] OR “Appliance, Orthodontic” OR “Appliances, Orthodontic” OR “Orthodontic appliance”  #9 (#1 OR #2 OR #3) AND #4 AND #8  #10 (#1 OR #2 OR #3) AND #5 AND #8  #11 (#1 OR #2 OR #3) AND #6 AND #8  #12 (#1 OR #2 OR #3) AND #7 AND #8 |

**Table S3.** Summary table of studies excluded in this scoping review because not relevant to the main subject.

| **Excluded Studies** | **Exclusion Reasons** |
| --- | --- |
| Gonçalves e Silva et al., 2013  [21] | Assess the effect of fixed OA on microbic composition |
| Low et al., 2010  [22] | Assess the effect of fixed OA on microbic composition |
| Leung et al., 2006  [23] | Assess the effect of fixed OA on microbic composition |
| Faccioni et al., 2019  [24] | Assess the effect of a removable OA |
| Cruz et al., 2021  [25] | Assess the effect of a removable OA |
| Dallel et al., 2020  [26] | Assess the effect of fixed OA on salivary parameters |
| Raducanu et al., 2022  [27] | Assess the effect of fixed OA on salivary parameters |
| Guler et al., 2014  [28] | Assess the effect of fixed OA on salivary parameters |
| Angelieri et al., 2010  [29] | Assess the effect of radiographs during FOT |
| Taubmann et al., 2021  [30] | Assess the effects of the resin-based adhesive used during FOT |
| Durgo et al., 2023  [31] | Assess the effect of fixed OA on the cells of the mucosa of gastrointestinal tract |

Abbreviations: FOT: Fixed Orthodontic Treatment; OA: Orthodontic Appliance

**Table S4.** Bias domains included in the ROBINS-I-tool

| **Domain** | **Related terms** | **Explanation** | |  |
| --- | --- | --- | --- | --- |
| *Pre-intervention* |  |  | | Pre-intervention or at-intervention domains for which risk of bias assessment is mainly distinct from assessments of randomized trials |
| Bias due to confounding | Selection bias *as it is sometimes used in relation to clinical trials* (and currently in widespread use within Cochrane); Allocation bias; Case-mix bias; Channelling bias. | Baseline confounding occurs when one or more prognostic variables (factors that predict the outcome of interest) also predicts the intervention received at baseline. ROBINS-I can also address time-varying confounding, which occurs when individuals switch between the interventions being compared and when post-baseline prognostic factors affect the intervention received after baseline. | |  |
| Bias in selection of participants into the study | Selection bias *as it is usually used in relation to observational studies and sometimes used in relation to clinical trials*; Inception bias; Lead- time bias; Immortal time bias. Note that this bias specifically excludes lack of external validity, which is viewed as a failure to generalize or transport an unbiased (internally valid) effect estimate to populations other than the one from which the study population arose. | When exclusion of some eligible participants, or the initial follow up time of some participants, or some outcome events, is related to both intervention and outcome, there will be an association between interventions and outcome even if the effects of the interventions are identical. This form of selection bias is distinct from confounding. A specific example is bias due to the inclusion of prevalent users, rather than new users, of an intervention. | |  |
| *At intervention* |  |  | |  |
| Bias in classification of interventions | Misclassification bias; Information bias; Recall bias; Measurement bias; Observer bias. | Bias introduced by either differential or non-differential misclassification of intervention status. Non-differential misclassification is unrelated to the outcome and will usually bias the estimated effect of intervention towards the null. Differential misclassification occurs when misclassification of intervention status is related to the outcome or the risk of the outcome, and is likely to lead to bias. | |  |
|  |  |  |  |  |

| *Post-intervention* |  |  | Post-intervention domains for which there is substantial overlap with assessments of randomized trials |
| --- | --- | --- | --- |
| Bias due to deviations from intended interventions | Performance bias; Time-varying confounding | Bias that arises when there are systematic differences between experimental intervention and comparator groups in the care provided, which represent a deviation from the intended intervention(s). Assessment of bias in this domain will depend on the type of effect of interest (either the effect of assignment to intervention or the effect of starting and adhering to intervention). |  |
| Bias due to missing data | Attrition bias; Selection bias *as it is sometimes used in relation to observational studies* | Bias that arises when later follow-up is missing for individuals initially included and followed (e.g. differential loss to follow-up that is affected by prognostic factors); bias due to exclusion of individuals with missing information about intervention status or other variables such as confounders. |  |
| Bias in measurement of outcomes | Detection bias; Recall bias; Information bias; Misclassification bias; Observer bias; Measurement bias | Bias introduced by either differential or non-differential errors in measurement of outcome data. Such bias can arise when outcome assessors are aware of intervention status, if different methods are used to assess outcomes in different intervention groups, or if measurement errors are related to intervention status or effects. |  |
| Bias in selection of the reported result | Outcome reporting bias; Analysis reporting bias | Selective reporting of results in a way that depends on the findings. |  |

**Table S5.** Bias Analysis of the studies included in this review using the ROBINS-I-tool for Observational Studies

| **Reference**  **First author  et al.**  **Year** | **D1** | **D2** | **D3** | **D4** | **D5** | **D6** | **D7** |  |
| --- | --- | --- | --- | --- | --- | --- | --- | --- |
| [32]  Natarajan et al.  2010 | 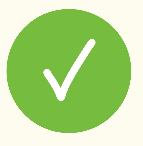 | 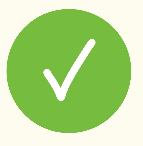 | 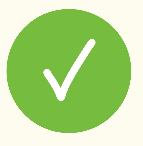 | 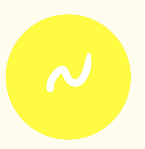 | 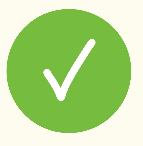 | 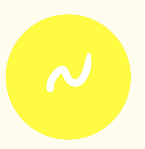 | 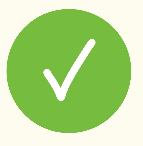 |  |
| [33]  Hafez et al.  2011 | 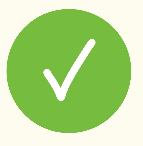 | 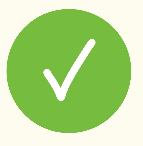 | 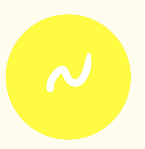 | 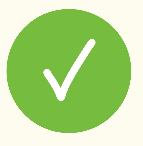 | 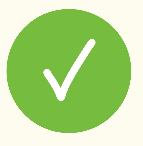 | 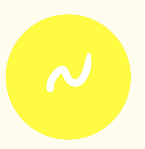 | 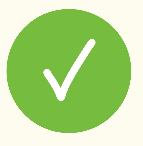 |  |
| [34]  Kapadia et al.  2018 | 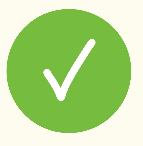 | 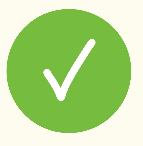 | 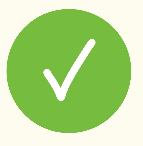 | 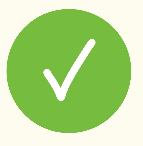 | 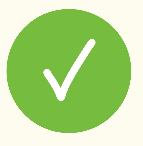 | 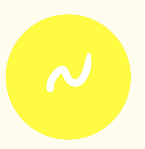 | 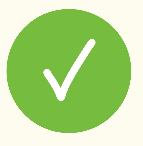 |  |
| [35]  Faccioni et al.  2003 | 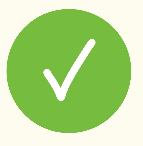 | 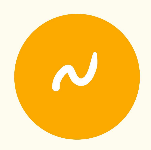 | 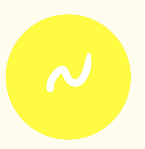 | 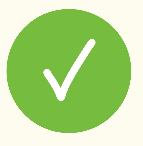 | 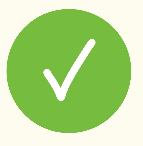 | 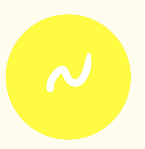 | 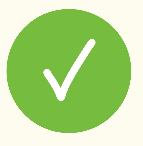 |  |
| [36]  Amini et al.  2008 | 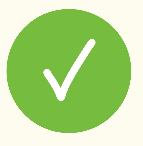 | 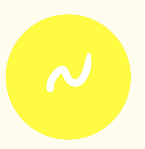 | 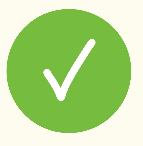 | 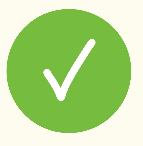 | 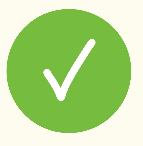 | 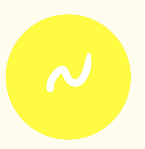 | 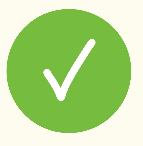 |  |
| [37]  Fernández-Miñano et al. 2011 | 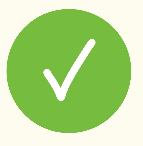 | 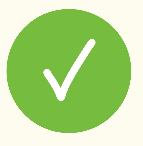 | 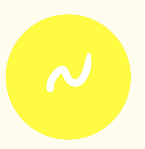 | 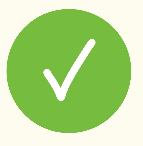 | 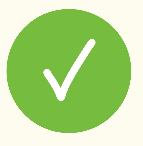 | 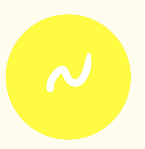 | 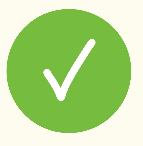 |  |
| [38]  Alsalhi et al.  2019 | 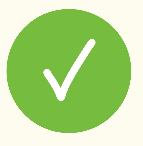 | 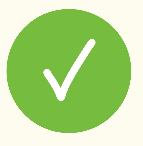 | 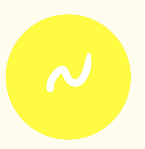 | 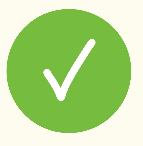 | 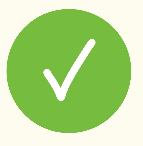 | 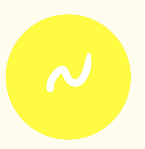 | 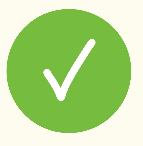 |  |
| [39]  Sampaio Mei et al.  2013 | 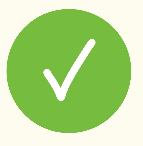 | 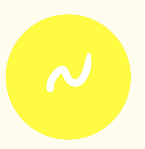 | 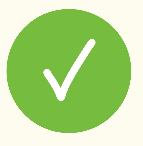 | 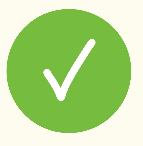 | 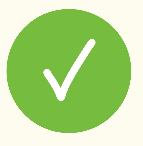 | 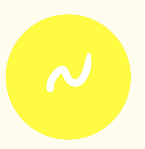 | 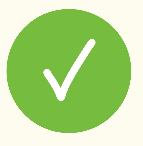 |  |
| [40]  Arruda et al.  2011 | 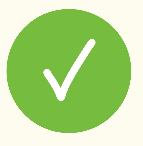 | 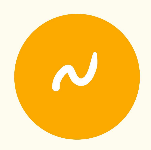 | 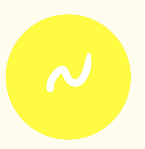 | 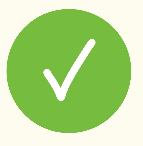 | 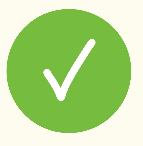 | 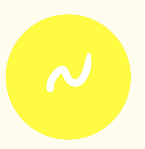 | 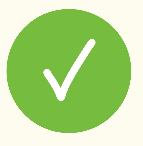 |  |
| [41]  Rafighi et al.  2020 | 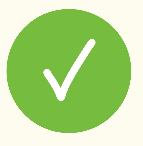 | 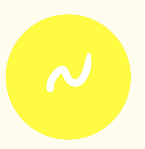 | 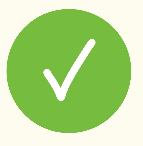 | 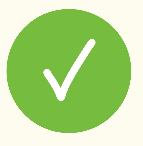 | 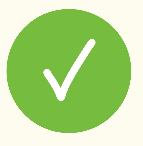 | 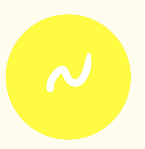 | 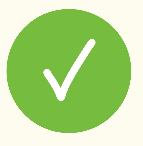 |  |
| [42]  Pereira et al.  2008 | 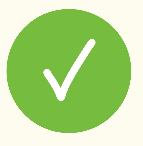 | 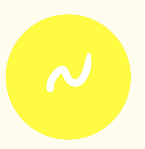 | 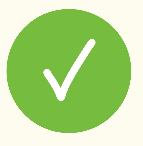 | 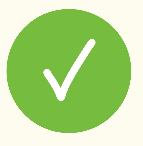 | 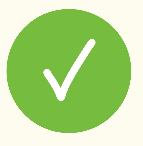 | 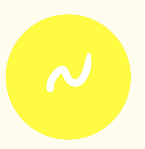 | 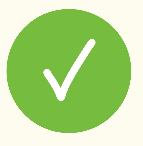 |  |
| [43]  Buczko et al.  2017 | | 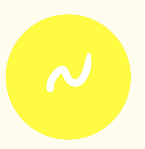 | 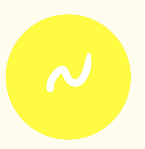 | 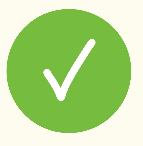 | 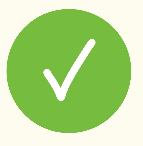 | 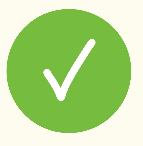 | 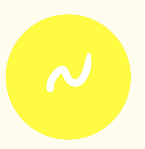 | 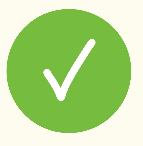 |
| [44]  Carrillo-Novia et al.  2006 | 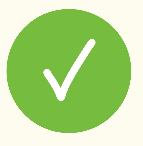 | 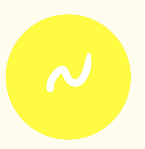 | 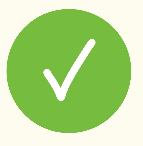 | 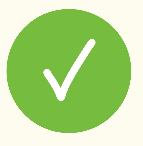 | 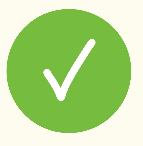 | 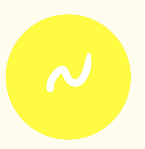 | 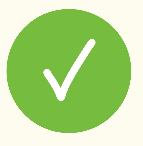 |  |
| [45]  Francis et al.  2017 | 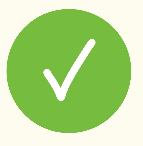 | 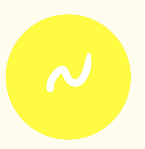 | 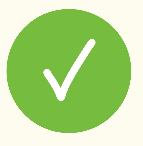 | 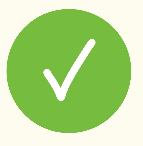 | 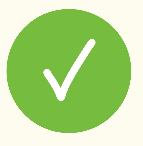 | 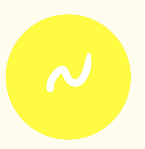 | 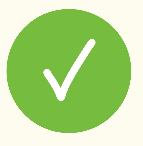 |  |
| [46]  Cunha et al.  2018 | 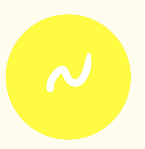 | 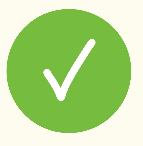 |  |  |  |  |  |  |
| [47]  Gonçalves et al.  2015 |  |  |  |  |  |  |  |  |
| [48]  Angelieri et al.  2011 |  |  |  |  |  |  |  |  |
| [49]  Flores-Bracho et al.  2019 |  |  |  |  |  |  |  |  |
| [50]  Westphalen et al.  2008 |  |  |  |  |  |  |  |  |

**Questions**
D1: Bias due to confounding
D2: Bias due to selection of participants
D3: Bias in classification of interventions
D4: Bias due to deviations from intended intervention
D5: Bias due to missing data
D6: Bias in measurement of outcomes
D7: Bias in selection of the reported results
**Possible Answers**
(1)Low risk of bias (the study is comparable to a well-performed randomized trial with regard to this domain): Green Symbol
(2)Moderate risk of bias (the study is sound for a non-randomized study with regard to this domain but cannot be considered comparable to a well-performed randomized trial): Yellow Symbol
(3)Serious risk of bias (the study has some important problems in this domain): Orange Symbol
(4)Critical risk of bias (the study is too problematic in this domain to provide any useful evidence on the effects of intervention): Red Symbol
(5)No information on which to base a judgement about risk of bias for this domain: No Symbol

**Table S6.** NHLBI Quality Assessment Tool for Observational Cohort and Cross-Sectional Studies

| **Reference**  **First author et al. Year** | **Q1** | **Q2** | **Q3** | **Q4** | **Q5** | **Q6** | **Q7** | **Q8** | **Q9** | **Q10** | **Q11** | **Q12** | **Q13** | **Q14** | **Total score** | **Quality rating** |
| --- | --- | --- | --- | --- | --- | --- | --- | --- | --- | --- | --- | --- | --- | --- | --- | --- |
| [32]  Natarajan et al.  2010 | Y | Y | Y | Y | Y | NA | Y | NA | Y | Y | Y | N | Y | NR | 10/14  71,43% | Fair |
| [33]  Hafez et al.  2011 | Y | Y | Y | Y | Y | NA | Y | NA | NA | Y | Y | N | Y | NR | 9/14  64,28% | Fair |
| [34]  Kapadia et al.  2018 | Y | Y | N | Y | N | Y | Y | NA | NA | Y | Y | N | Y | NR | 8/14  57,14% | Fair |
| [35]  Faccioni et al.  2003 | Y | N | Y | Y | N | N | Y | NA | NA | N | Y | N | Y | NR | 6/14  42,86% | Poor |
| [36]  Amini et al.  2008 | Y | Y | Y | Y | N | N | N | NA | NA | N | Y | N | Y | NR | 6/14  42,86% | Poor |
| [37]  Fernández-Miñano et al. 2011 | Y | Y | Y | Y | N | Y | Y | NA | NA | N | Y | N | Y | NR | 8/14  57,14% | Fair |
| [38]  Alsalhi et al.  2019 | Y | Y | Y | N | N | N | Y | NA | NA | N | Y | Y | Y | NR | 6/14  42,86% | Poor |
| [39]  Sampaio Mei et al.  2013 | Y | Y | Y | Y | Y | Y | Y | NA | NA | Y | Y | N | Y | NR | 10/14  71,43% | Fair |
| [40]  Arruda et al.  2011 | Y | N | Y | Y | Y | NR | Y | NA | NA | N | Y | N | Y | NR | 7/14  50,00% | Fair |
| [41]  Rafighi et al.  2020 | Y | Y | Y | Y | Y | N | Y | NA | NA | Y | Y | N | Y | NR | 9/14  64,28% | Fair |
| [42]  Pereira et al.  2008 | Y | Y | Y | Y | N | Y | Y | NA | NA | Y | Y | N | Y | NR | 9/14  64,28% | Fair |
| [43]  Buczko et al.  2017 | Y | Y | Y | Y | N | Y | Y | NA | NA | Y | Y | Y | Y | NR | 10/14  71,43% | Fair |
| [44]  Carrillo-Novia et al.  2006 | Y | Y | Y | Y | Y | Y | Y | NA | NA | Y | Y | N | Y | Y | 11/14  78,57% | Good |
| [45]  Francis et al.  2017 | Y | Y | Y | Y | N | N | Y | NA | NA | Y | Y | N | Y | NR | 8/14  57,14% | Fair |
| [46]  Cunha et al.  2018 | Y | Y | Y | Y | N | Y | Y | NA | NA | Y | Y | Y | Y | NR | 10/14  71,43% | Fair |
| [47]  Gonçalves et al.  2015 | Y | Y | Y | Y | Y | Y | Y | NA | NA | Y | Y | Y | Y | NR | 11/14  78,57% | Good |
| [48]  Angelieri et al.  2011 | Y | N | Y | N | N | Y | Y | NA | NA | Y | Y | N | Y | Y | 8/14  57,14% | Fair |
| [49]  Flores-Bracho et al.  2019 | Y | Y | Y | Y | Y | N | Y | NA | NA | N | Y | Y | Y | Y | 10/14  71,43% | Fair |
| [50]  Westphalen et al.  2008 | Y | N | Y | Y | N | Y | N | NA | NA | N | Y | N | Y | NR | 6/14  42,86% | Poor |

Q1: Was the research question or objective in this paper clearly stated?, Q2: Was the study population clearly specified and defined?, Q3: Was the participation rate of eligible persons at least 50%?, Q4: Were all the subjects selected or recruited from the same or similar populations (including the same time period)? Were inclusion and exclusion criteria for being in the study prespecified and applied uniformly to all participants?, Q5: Was a sample size justification, power description, or variance and effect estimates provided?, Q6: For the analyses in this paper, were the exposure(s) of interest measured prior to the outcome(s) being measured?, Q7: Was the timeframe sufficient so that one could reasonably expect to see an association between exposure and outcome if it existed?, Q8: For exposures that can vary in amount or level, did the study examine different levels of the exposure as related to the outcome (e.g., categories of exposure, or exposure measured as continuous variable)?, Q9: Were the exposure measures (independent variables) clearly defined, valid, reliable, and implemented consistently across all study participants?, Q10: Was the exposure(s) assessed more than once over time?, Q11: Were the outcome measures (dependent variables) clearly defined, valid, reliable, and implemented consistently across all study participants?, Q12: Were the outcome assessors blinded to the exposure status of participants?, Q13: Was loss to follow-up after baseline 20% or less?, Q14: Were key potential confounding variables measured and adjusted statistically for their impact on the relationship between exposure(s) and outcome(s)?; Total Score: Number of yes; CD: cannot be determined; NA: not applicable; NR: not reported; N: no; Y: yes. Quality Rating: Poor <50%, Fair 50–75%, Good

**Table S7**. Baseline characteristics of the patients, study design inclusion criteria and exclusion criteria of the studies considered in this review.

| **Reference**  **Authors**  **Year of Publication**  **Origin of the Research**  **Study Design** | **Inclusion Criteria** | **Exclusion Criteria** |
| --- | --- | --- |
| [32]  Natarajan et al.  2010 | - At least 18 months of Orthodontic Therapy - Permanent Dentition | - Previous Fixed Orthodontic Treatment - Systemically Debilitating Diseases or Syndromes - Removable Prostheses - Mutagenic Hazards Exposure (Smoking, Drinking, Illness-related) - Assumption of Antibiotics, Steroids - Usage of Alcohol-based Mouthwashes - Extraoral or Palatal/Lingual Appliances - Presence of Sharp Edges (Restorations or Prostheses) |
| [33]  Hafez et al.  2011 | - Good Oral Hygiene | - Previous Fixed Orthodontic Treatment - Soft Tissues Lesions - Metal Allergy - Occupational Exposition - Assumption of Medications or Dietary Supplements - Radiographic Examinations in the previous 6 months |
| [34]  Kapadia et al.  2018 | - N.R. | - Age > 38 years - Previous Fixed Orthodontic Treatments - Metal Allergy - Smoking - Metal Restorations |
| [35]  Faccioni et al.  2003 | - Permanent dentition | - Dental Restorations |
| [36]  Amini et al.  2008 | - N.R. | - Systemic Diseases - Smoking - Assumption of Medications related to Mucosal Changes - Metal Restorations or Intraoral Piercings |
| [37]  Fernández-Miñano et al. 2011 | - Good Oral Health - Need of orthodontic treatment at both arches - Ability to carry out correct oral hygiene procedures | - Systemic Diseases - Cavities or repairing treatment - Treatments that could generate psychological alteration or difficulties in everyday relationships |
| [38]  Alsalhi et al.  2019 | - Good oral Hygiene | - Previous Fixed Orthodontic Treatments - Systemically Debilitating Diseases - Soft Tissues Lesions - Metal Allergy - Local Irritating Factors - Traumatizing Behaviors (e.g. cheek biting) |
| [39]  Sampaio Mei et al.  2013 | - N.R.. | - Systemically Debilitating Diseases - Soft Tissues Lesions - Removable Prostheses - Smoking, Drinking - Assumption of Antibiotics, Steroids - Usage of Mouthwashes |
| [40]  Arruda et al.  2011 | - At least 24 months of Orthodontic Therapy - Ni-Cr Appliances | - Anemia - Smoking, Drinking |
| [41]  Rafighi et al.  2020 | - N.R.. | - Systemically Debilitating Diseases - Smoking, Drinking - Assumption of Antibiotics or Steroids - Usage of Mouthwashes - Local Irritating Factors - Severe Crowding |
| [42]  Pereira et al.  2008 | - N.R.. | - Systemic Diseases - Soft Tissues Lesions - Sharp Edges - Smoking, Drinking - Assumption of Antibiotics or Steroids - Usage of Alcohol-Based Mouthwashes |
| [43]  Buczko et al.  2017 | - General Good Health - Good Oral Hygiene | - Smoking - Assumption of Antibiotics or Steroids 6 months prior to sample collection - Systemic Disease - Oral Infection |
| [44]  Carrillo-Novia et al.  2006 | - Clinically Healthy Mucosa - Ni-Cr or Cr-Cb Appliances | - Systemic Diseases, Illnesses related to Genetic damage - Metal Allergy - Metal Restorations - Addiction or Assumption of Medications or Dietary Supplements |
| [45]  Francis et al.  2017 | - General Good Health - Good Oral Hygiene | - Smoking, Drinking - Dental Restorations - Usage of Oral Antiseptic Solutions - Loss of at least 4 teeth - Caries - Aphtous Stomatitis - Skin Reactions |
| [46]  Cunha et al.  2018 | - Mixed Dentition - Posterior Crossbite | - Previous Fixed Orthodontic Treatment - Systemic Diseases, Syndromes, Illnesses related to Genetic Damage - Metal Allergy - Prostheses or Restorations - Smoking, Drinking - Exposition to chemicals or radiation - Assumption of Antibiotics or Steroids - Usage of Alcohol-based Mouthwashes - Periodontitis or Carious Lesions |
| [47]  Gonçalves et al.  2015 | - Good General Health | - Previous Orthodontic Treatments with Metallic Appliances - Metal Restorations - Smoking, Drinking |
| [48]  Angelieri et al.  2011 | - Good General Health | - N.R.. |
| [49]  Flores-Bracho et al.  2019 | - Permanent Dentition | - Previous Fixed Orthodontic Treatments - Systemic Diseases, Illnesses related to Genetic Damage - Metal Allergy - Smoking, Drinking, Drug Addiction - Assumption of Antibiotics or Steroids - Usage of Alcohol-based Mouthwashes - Exposition to Chemicals or Radiations - Amalgam Restorations - Caries, Periodontitis |
| [50]  Westphalen et al.  2008 | - General Good Health | - Smoking, Drinking - Illnesses related to genetical damage |

Abbreviations: N.R.: Not Reported

**Table S8**. Sample collection, treatment and assessment of the studies included in this review.

| **Reference**  **Authors**  **Year of Publication** | **Sample Collection and Treatment** | **Sample Assessment Method** | |
| --- | --- | --- | --- |
| [32]  Natarajan et al.  2010 | Mouth Rinsing with distilled Water for several times, Scraping with a metal spatula (inside part of the lips and buccal mucosa), Transfer to a Glass, Fixation with Absolute Alcohol, Hydration with Water, Staining with the Papanicolaou Technique | - Light Microscopy | |
|  | Mouth Rinsing with distilled Water for several times, Scraping with a cytological brush (inside part of the lips and buccal mucosa), Transfer to a centrifuge tube containing distilled Water, Centrifuge, Addition of 1 drop of pure Nitric Acid (only to 33% of the solution) | - Coupled plasma-mass Spectrometry | |
| [33]  Hafez et al.  2011 | Scraping with a wooden stick, Placement in a phosphate-buffered saline solution, Storage on ice and Transfer to the Laboratory | - Trypan Blue Dye Test - Comet Assay - Graphite Furnace Atomic Absorption Spectrometry | |
| [34]  Kapadia et al.  2018 | Mouth Rinsing with distilled Water twice, Scraping with an interdental Brush (buccal mucosa), Centrifugation of the cells, Transfer to a Phosphate-buffered Saline Solution at a pH of 7,4, Filtration through a Polyamide Gauze, Centrifugation, re-Suspension in 1mL of Phosphate.Buffered Saline Solution (PBS) | - Light microscopy - Comet assay - Atomic Absorption Spectrometry | |
| [35]  Faccioni et al.  2003 | Mouth rinsing with tepid Water many times; Scraping with an interdental Brush; Stirring in a 5mL PBS, Centrifugazion, Suspension in PBS, Filtration through Polyamide Gaze | | |
|  | Centrifugation; Suspension in RPMI-1640 (Life Technology, Milan, Italy); Staining with Trypan Blue technique | - Light microscopy | |
|  | Centrifugation; Suspension in RPMI-1640 (Life Technology, Milan, Italy); Addition of 75microL of low-melting-point-agarose to 10microL of cell suspension; Transfer to a slide, Lysis at pH 10; Electrophoresis; Neutralization; Drying with 100% ethanol; Staining with Ethidium Bromide | - Comet assay | |
|  | Centrifugation; Suspension in RPMI-1640 (Life Technology, Milan, Italy); Addition of 2mL of 0,5% Nitric Acid to 1mL of Cell Suspension; Diluition with deionized distilled Water | - Coupled plasma-mass spectrometry | |
| [36]  Amini et al.  2008 | Mouth Rinsing; Scraping with an interdental Brush (bilateral cheek mucosa); Transfer to a tube containing 5mL of Phosphate-buffered Saline Solution; Diluition in Water, Acidification with Nitric Acid at 60°C for 10 minutes | - Graphite-furnace atomic absorption spectrophotometry | |
| [37]  Fernández-Miñano et al. 2011 | Mouth rinsing with temperate water, Cell collection with Interproximal brushes in the internal part of the cheek, Agitation in a 15mL tube containing PBS, Centrifugation at 1.5000 rpm and 15°C for 10 minutes, Resuspension in PBS | | |
|  | Positioning of the suspension on two slides: the bottom with normal melting agarose, the top with low-melting-point-agarose, Treatment with H_2_O_2_, Lysis with Trypsin in PBS, Washing, Treatment with Proteinase K, Rinsing, Positioning in EDTA Lysis solution, Washing, Positioning in an electrophoresis chamber at pH 9, Staining with ethidium bromide, Observation under fluorescence microscopy | - Comet assay | |
|  | Treatment of 100 microL of cell suspension with 0,5% nitric acid, Addition to 10 mL ultrapure water, Conservation at -80°C, Analyses using inductively coupled plasma-mass spectrometer | - Coupled plasma-mass spectrometry | |
| [38]  Alsalhi et al.  2019 | Mouth Rinsing with water, Scraping with a Wooden tongue blade, Transfer to a glass, Fixation with 99,9% Alcohol, Staining with the Papanicolau Technique | - Light microscopy | |
| [39]  Sampaio Mei et al.  2013 | Brush scraping, Placement in the UCM (Universal Collection Medium) solution, Transfer to a polycarbonate membrane, Staining with the Papanicolaou technique | - Light microscopy | |
| [40]  Arruda et al.  2011 | Brush scraping, Placement in the UCM solution, Transfer to a polycarbonate membrane, Attachment to a Glass Slide, Fixation with 99,9% Alcohol, Staining with the Papanicolaou technique | - Light microscopy | |
| [41]  Rafighi et al.  2020 | Mouth Rinsing with Water, Scraping with a Cytological Brush (lower lip mucosa), Transfer to a glass slide, Fixation with 96% Alcohol, Storage at 4°C, Staining with Papanicolaou technique | - Light microscopy | |
| [42]  Pereira et al.  2008 | Mouth Rinsing with Water, Collection and Suspension of the cells with the DNA-Citoliq System kit (Digene Brasil LTDA, Sao Paulo, Brasil) in the UCM, Staining with the Papanicolaou technique | - Light microscopy | |
| [43]  Buczko et al.  2017 | Mouth Rinsing with distilled water; Scraping with a spatula, Transfer to a Glass Slide, Fixation with 96% ethanol, Staining with May-Grunwald-Giemsa staining | - Light microscopy - Graphite atomic absorption spectrometry | |
| [44]  Carrillo-Novia et al.  2006 | Mouth Rinsing with purified Water for 30s, Mucous Curettage with a Cytological Brush, Transfer to a Fixative Solution, Agitation of the Sample Storage at 4°C, Addition of 3mL gradient solution to 3mL sample, Placement in a Falcon Tube, Centrifuge, Transfer to the microscope slide, Fixation with 96% ethanol, Staining with Papanicolaou technique | - Light microscopy | |
| [45]  Francis et al.  2017 | Mouth Rinsing with distilled Water several times; Scraping with a Cement Spatula (middle cheek mucosa); Transfer to a Glass Slide; Fixation; Staining with Papanicolaou technique | - Light microscopy | |
| [46]  Cunha et al.  2018 | Mouth Rinsing with 0,9% Sodium Chloride Solution twice (for one minute each), Scraping (bilateral cheek mucosa), Transfer to a plastic tube containing 0,9% Sodium Saline Solution, Storage at 4°C, Centrifugation, Suspension in a Fixative Solution, Addition of five drops of Dimethyl Sulfoxide, Repetition of the last 3 passages, Transfer to a Glass Slide, Dried ad room temperature for 24 hours, Staining with the Feulgen/Fast Green method | - Light microscopy | |
| [47]  Gonçalves et al.  2015 | Mouth Rinsing with distilled Water several times, Scraping with a Cytological Brush (bilateral cheek), Transfer to a Falcon tube containing 20 mL buffer, Storage at 4°C. | | |
|  | Nuclear Alterations  Air-Drying for 10 minutes, Fixation with methanol:acetic acid (3:1), Staining with Feulgen technique | | - Light Microscopy |
|  | DNA damage Centrifugation, re-Suspension in 1 mL buffer, Transfer to Eppendorf tube, Centrifugation, Addition of 0,25% Trypsin Solution, Treatment with Proteinase K, Mixture with 0,75% low-melting point Agarose, Transfer to a Glass Slide, Setting at 4°C, Incubation in ice-cold Lysis Solution at 4°C for 1 week, Electrophoresis, Neutralization, Washing with distilled Water, Staining with AgNO_3_ | | - Comet assay |
| [48]  Angelieri et al.  2011 | Mouth Rinsing with tap Water, Scraping with a moist Wooden Spatula (bilateral cheek mucosa), Transfer to a tube containing Saline Solution, Centrifugation, Fixation with methanol:acetic acid (3:1), Transfer to a Glass Slide, Staining with Feulgen/Fast Green method | - Light microscopy | |
| [49]  Flores-Bracho et al.  2019 | Mouth Rinsing with Saline Solution for 2 minutes, twice; Scraping with a Toothbrush (bilateral cheek mucosa), Transfer to a plastic Tube containing 5mL of 0,9% Sodium Chloride Solution; Storage at 4°C; Transfer to the Laboratory; Centrifugation; Removal of the Supernatant part with a Pipette; Addition of Methanol:Acetic Acid (3:1) and Dimethyl Sulfoxide; Centrifugation; Removal of the Supernatant Part; Transfer to a Slide; Drying at room temperature for 24h; Staining with Feulgen/Fast Green | - Light microscopy | |
| [50]  Westphalen et al.  2008 | Mouth rinsing with distilled water several times, Gentle brushing of the inside part of the lower lip with a Cytological Brush, Stirring in 50mL plastic tubes containing 20mL PBS, Washing and Centrifugation at 1.500rpm for 10 minutes at room temperature, Resuspension in PBS | | |
|  | Dropping of cell suspension on pre-warmed Slides (37°C), Air-dry, Fixation in Methanol 80% at 0°C for 20 minutes, Staining with May-Grunwald-Giemsa, Examination under Light Microscopy | | - Light Microscopy |
|  | Mixing of 10microL of cell suspension with 75microL of low-melting-point-agarose, Addition to a agarose-pre-coated slide, Lysis at 10 pH, Placement in a alkaline electrophoresis chamber, Neutralization, Fixation, Staining with silver nitrate, Examination under Light Microscopy at 1.000X magnification | | - Comet assay |

Abbreviations: P.B.S., Phosphate buffer Saline Solution; PCR , Polymerase Chain Reaction; U.C.M., Universal Collection Medium

**Table S9**. Staining techniques and number of cells assessed per patients in cytological and nuclear assessment, and for BCA.

| **Study** | **Staining technique** | **How many cells**  **have been assessed** | **Sample Assessment Method** |
| --- | --- | --- | --- |
| [32]  Natarajan et al.  2010 | Papanicolaou | 1000/sample | Light microscopy |
| [33]  Hafez et al.  2011 | Trypan Blue | N.R. | Light microscopy |
|  | Ethium Bromide | 50/sample | BCA |
| [34]  Kapadia et al.  2018 | Papanicolaou, Trypan Blue | N.R. | Light microscopy |
|  | N.R. | N.R. | BCA |
| [35]  Faccioni et al.  2003 | Trypan Blue | N.R. | Light microscopy |
|  | Ethium Bromide | 100/sample | BCA |
| [37]  Fernández-Miñano et al.  2011 | Ethium Bromide | 200/sample | BCA |
| [38]  Alsalhi et al.  2019 | Papanicolaou | Observation of 5 different  areas of 1cm^2^ | Light microscopy |
| [39]  Sampaio Mei et al.  2013 | Papanicolaou | 50/sample | Light microscopy |
| [40]  Arruda et al.  2011 | Papanicolaou | A total of 3300 on a  group of 20 subjects | Light microscopy |
| [41]  Rafighi et al.  2020 | Papanicolaou | A total of 4650 on a  group of 31 subjects | Light microscopy |
| [42]  Pereira et al.  2008 | Papanicolaou | 50/sample | Light microscopy |
| [43]  Buczko et al.  2017 | May-Grunwald-Giemsa | 50/sample | Light microscopy |
| [44]  Carrillo-Novia et al.  2006 | Papanicolaou | 1000/sample | Light microscopy |
| [45]  Francis et al.  2017 | Papanicolaou | Cells seen on a zigzag movement of the whole slide | Light microscopy |
| [46]  Cunha et al.  2018 | Feulgen/Fast Green | 1000/sample | Light microscopy |
| [47]  Gonçalves et al.  2015 | Feulgen/Fast Green | 1000/sample | Light microscopy |
|  | Silver Nitrate | 100/sample | BCA |
| [48]  Angelieri et al.  2011 | Feulgen/Fast Green | 2000/sample | Light microscopy |
| [49]  Flores-Bracho et al.  2019 | Feulgen/Fast Green | 2000/sample | Light microscopy |
| [50]  Westphalen et al.  2008 | May-Grumwald-Giemsa | 1000/sample | Light microscopy |
|  | Silver Nitrate | 50/sample | BCA |

Abbreviations: BCA: Buccal Comet Assay, N.R.: Not Reported

**Table S10.** Evidence of studies included in this review.

| **Reference**  **Authors**  **Year of Publication** | **Study Design and Aim** | **Methods** | **Results** | **Conclusions** |
| --- | --- | --- | --- | --- |
| [32]  Natarajan et al.  2010 | A Prospective Case-Control Observational Study aiming to evaluate the possible genotoxic damage to the oral mucosal cells in healthy patients undergoing orthodontic treatment with fixed appliance; evaluate the persistence of these genotoxic changes at 1 month after debonding; evaluate the metal ion content in the oral mucosal cells, in order to establish a correlation between this and the genotoxic changes | - 20 subjects undergoing fixed orthodontic therapy were compared with 20 control subjects. The time points for the assessment were set at debonding (T0) and 30 days after (T1). - The smears were assessed for the presence of micronuclei (under light microscopy) and for the nickel and chromium cellular content (using the coupled plasma-mass spectrometry) | - The mean MN frequency at T0 was higher in the experimental group than in the control group (p<0.05) - The change in the mean MN frequency was significant between T0 and T1, only in the experimental group (p<0.0001) - The only significant result concerning metal cellular content is a higher level of chromium at T1 in the experimental group, when compared to the control group. - There is a significant correlation in the presence of MN and in the chromium ion content at T1 in the experimental group (p=0.03). No other significant correlations have been found. | Nickel and chromium alloys contained in the orthodontic appliances can emit sufficient metal ions, in order to induce localized genotoxic effects. These are reversible when the source of the ions is removed. |
| [33]  Hafez et al.  2011 | A Prospective Case-Control Longitudinal Study aiming to test the biocompatibility of fixed orthodontic appliances | - 28 subject undergoing fixed orthodontic treatment were compared with 18 subject not receiving the treatment. The case group was sub-divided in four groups according to the combination of brackets and archwires. The time points for the assessment were set before the beginning of the treatment (T0), 3 months (T1) and 6 months (T2) after the application of the appliances. - The smears were analyzed for the Cell viability (using the Trypan blue exclusion test), the DNA damage and frequency (using the Alkaline comet assay), the Nickel and Chromium content (using the Graphite Furnace Atomic Absorpion Spectrometry) | - In the test group, the viability decreased significantly only at T2, when compared to T0 (p=0,009) - The nickel and the chromium content increased significantly both at T1 and at T2, when compared to T0; p value was 0.000 in all these measurements - The Composite Score, evaluating the DNA damage, is decreased significantly only at T2, when compared to T0 (p=0,009) - The damage frequency showed no significant differences at all the time points (p>0.05) - In the control group, the Composite score showed a statistically significant diminution at T1, when compared to T0 (p=0.000). - The damage frequency decreased at T1, when compared to T0 (p=0.003) - The relative risk showed significant differences between the control and case groups at T1 for the cellular Chromium content (p=0.038), the Composite Score (p=0.017) and damage frequency(p=0.04). At T2 there has not been found a significant difference between control and case groups for any of the variables (p>0.05) - When comparing the viability in the 4 subgroups, the significant differences were found in the group 1, both at T1 and at T2 ;when compared to T0 (both with p=0.035), and in the group 4, both at T1 (p=0.033) and at T2 (p=0.011), when compared to T0. The cellular Nickel and Chromium content augmented significantly (p<0.05) at all time points for groups 1 and 2, and only at T1 for group 3 - The composite score diminished significantly at T1 for group 2 and at T2 for groups 2 and 4 (p<0.05), when compared to T0. - Damage frequency increased both in group 3 and 4 at all time-points (p<0.05) | The epithelial cells of the fixed orthodontic patients showed significant increases in nickel and chromium content, and significant decreases in the viability and in the DNA damage. When compared with the control group, the case group showed significant changes for the chromium content and DNA damage only at T1, indicating there might be a tissue reparative factor influencing these results.  The least biologically damaging components were stainless steel brackets and stainless steel archwires, while the most damaging ones were titanium brackets and nickel-titanium archwires |
| [34]  Kapadia et al.  2018 | Prospective Cohort Observational study aiming to Assess the presence of metal ions and DNA damage in cells of the buccal mucosa in subject scheduled to undergo fixed orthodontic tratment | - 80 subject receiving fixed orthodontic therapy have been assessed at five different time-points: before starting the therapy (T0), five months (T1), 10 months (T2), 15 months (T3) and 20 months (T4) after the insertion of the appliance. - The smears were assessed for the Viability (using the Trypan blue dye exclusion test), the DNA damage (using the comet assay and describing it through four parameters: mean length of the tail, percentage of DNA in tail, Olive tail movement and head diameter) and the nickel, chromium and zinc content (using the atomic absorption spectrometry) | - Viable cells level decreased in a non- significant way along all the time-points (p=0.07). - Levels of all the metal ions analyzed increased among all the time-points significatively (p=0.02, p=0.01, p=0.02 for Ni, Cr and Zn levels, respectively). - All the four parameters representing DNA damage decreased significantly among all the four points (p<0.02), apart from the olive tail movement, that increased non-significantly along all the time-points (p=0.09) | Patients receiving fixed orthodontic treatment present increasing levels of metal ions, though still remaining in the physiological dietary range. DNA damage occurs in these patients, indicating that timely checking can be a recommendable procedure. |
| [35]  Faccioni et al.  2003 | Cross-Sectional Case-Control Observational Study aiming to examine the concentration of metal ions in oral mucosa cells, the biocompatibility of orthodontic appliances and the possible DNA damage in the buccal cells by metal ion release | - 55 patients receiving fixed orthodontic therapy were compared with 30 controls, in a period included between 2-4 years after the placement of the appliance (T0). The smears were analyzed for cell viability, apoptosis, number of comets (all using alkaline comet assay), and for cobalt and nickel cellular content (using coupled plasma-mass spectrometry) | - Cytotoxicity in the experimental group has been showed, with significant increased comets (p=0.0047), increased apoptosis (p=021) and decreased viability (p=0.001). Comet assay also showed significant increase in tail moment (p<0.0001) and tail length increase (p=0.0001). - Both cobalt and nickel levels were significantly higher in the experimental group (p<0.001) | Nickel and cobalt can produce DNA breaks in cells of the oral epithelium. Loss of repair capacity could be an initiating event of adverse biological effects |
| [36]  Amini et al.  2008 | Cross-Sectional Case-Control Longitudinal Study Compare the content of nickel, chromium and cobalt in oral mucosa cells in young patients with and without orthodontic appliances | - 30 fixed orthodontic therapy were compared with 30 control. The smears, collected at around 16 month after appliances installation (T0), were assessed for nickel, chromium and cobalt cellular content (using graphite-furnace atomic absorption spectrophotometry) | - Both nickel, both chromium and cobalt levels in the test group were higher than in the control, but only nickel content changed significantly (p=0.003) (chromium and cobalt p>0.05) | Nickel level increase significantly in patients with fixed appliances. Chromium and cobalt levels don’t do the same. |
| [37]  Fernández-Miñano et al. 2011 | Prospective Observational Cohort Study aiming to assess DNA damage and cellular metal content in fixed orthodontic patients, before and after the beginning of the therapy | - 15 patients undergoing fixed orthodontic therapy were divided in three groups: group A (stainless steel appliance), group B (titanium appliance), group C (nickel-free appliance). The smears were collected before (T0) and 30 days after (T1) the placement of the appliance - The smears were assessed for the presence of DNA damage (under fluorescence microscopy) and for the metal cellular content (using inductively coupled plasma-mass spectrometry) | - Group A showed higher levels of Ti, M and Fe; none of them was significantly higher than that of T0 - Group B showed non-significant higher levels of Ti and M, when compared with T0 - Group C showed non-significant higher levels of Cr, Mn and Fe, when compared with T0 - DNA damage measured with the alkaline comet assay was significantly higher in the groups A and C at T1, when compared with T0 | Titanium brackets and tubes showed to be more compatible than stainless steel and nickel-free alloys |
| [38]  Alsalhi et al.  2019 | A Cross-Sectional Observational Case-Control study aiming to assess the morphological changes of oral mucosal epithelial cells in contact with fixed orthodontic appliances using exfoliated cytology | - 50 patients undergoing orthodontic fixed treatment were compared with 50 individuals not going under that treatment. The time point for assessment was between 3 and 6 months after the application of orthodontic appliances (T0). - The examination of the smears consisted in the assessment of cytomorphological changes in the smears analyzed using a light microscope | - There is a significant increase (p<0,001) in the number of cells as well as deviation characteristics from normal morphology in orthodontic patients as compared to control subjects. - No correlation between age, gender or period of treatment and cytomorphological chan-ges was observed | Epithelial cells showed some morphological changes in patients with orthodontic appliances, which represent an adaptive response |
| [39]  Sampaio Mei et al.  2013 | A Prospective Observational Cohort Study aiming to evaluate the effects of fixed orthodontic appliances on the epithelial cells of the buccal mucosa in the form of morphometric and morphological alterations | - 20 participants undergoing fixed orthodontic treatment were assessed at three time points: before the installation of the appliances (T0), 30 days after the installation (T1) and 30 days after the removal (T2). Each patient had elastic ties on the right side of the appliance and metal ties on the left side. - The examination of the smears included a cytomorphological and cytomorphometric assessment, using a light microscope | - There is a significant reduction in NA values (p<0,05) and an increase in CA values (p<0,05) between T0 and T1, for both metal and elastic ties group - There is a significant increase in NA values (p<0,05) and a reduction in CA values (p<0,05) between T1 and T2, for both metal and elastic ties group - The is not a significant change in the NA and CA values (p>0,05) between T0 and T2, only in the elastic ties group - The NA/CA ratio shows there is not any statistically significant group differences at any of the experimental periods | The orthodontic appliance causes adaptive cell changes characterized by an increased level of keratinization of the oral mucosa, with a tendency to return to the normal state after removal off the mechanical stimulus of the orthodontic device |
| [40]  Arruda et al.  2011 | A Cross-Sectional Cohort Observational Study aiming to assess the effect effect of friction of brackets and tubes on the epithelial cells of clinically normal oral mucosa using cytological and cytomorphometrical techniques | - The oral mucosa of 22 patients undergoing fixed orthodontic treatment was assessed in three parts: (I) part contacting the brackets, (II) part contacting the tube, (III) part not contacting the appliance. The time point for the collection was set at any time starting from 2 years after positioning the appliance (T0). - The examination of the smears included a cytomorphometrical and cytomorphological assessment under light microscopy | - There is a statistically significant reduction of NA of group (I) and (II) when compared to group (III) (p<0,01) - There is a difference between the change in the NA of the mucosa in contact with mandibular incisor brackets, but it is not statistically significant (p>0,05) - There is a significant change in the CA among the different groups (p<0,01) - There is no statistically significant difference among the NA/CA ratio of the three groups (p>0,05) - The main alterations found in the smears are class I by Papanicolau classification system, while the classes II are more common in the mucosa in contact with the band. This difference is not statistically significant (p>0,05) - There is a statistically significant predominance of nucleated cells in the superficial layer of the mucosa of group (II) (p=0,0097) | The main preclinical alteration s in the epithelial cells of oral mucosa, cause by orthodontic appliances, are reduction of the NA, cell keratinization and inflammatory features, especially in the parts of the mucosa in contact with the band tube |
| [41]  Rafighi et al.  2020 | Prospective Cohort Observational Study analyzing the changes in the lower lip oral mucosa in contact with orthodontic appliances on the day of debonding and after 30 and 60 days | - 31 fixed orthodontic patients have been assessed at three time points: just after debonding (T0), 30 days after (T1) and 60 days after (T2). - The smears were assessed for cytomorphometry and cytomorphology (both under light microscopy) | - The values of mean NA and CA increased significantly from T0 to T2 (p=0.000). - The NA/CA ratio did not change significantly (p=0.25). - The only Papanicolau class found in the smears is the class I. - The number of superficial cells decreased from T0 to T2, while the number of intermediate cells increased at the same time points. Nonetheless, the only significant change was found in the number of superficial cells (p=0.02) | Orthodontic appliances cause an increase in keratinization of the lower lip mucosa, considerable as an adaptative response to the mechanical stimulus. No reactive or dysplastic response has been found after the removal of the appliance. |
| [42]  Pereira et al.  2008 | Prospective Cohort Observational Study aiming to assess the buccal mucosa epithelial cells adjacent to metal and ceramic brackets at three time points: baseline, 60 days after placements, 30 days after removal | - 21 patients undergoing fixed orthodontic therapy were assessed at three different time-points: baseline (T0), 60 days after placement (T1) and 30 days after removal (T2). - The smears were assessed for cytomorphometry and cytomorphology (both using light microscopy) | - Between T0 and T1, NA and N/C ratio decreased, while CA increased, all of them significantly (p<0.01). Metal brackets caused significantly lower NA and N/C ratio (p<0.01) and non-significantly higher CA, when compared with ceramic brackets. - Between T1 and T2, metal brackets patients still showed lower NA and N/C ratio and higher CA, when compared with T0 (p<0.01), despite fewer changes when compared to T1 (p<0.01). - Ceramic brackets patients’ cells turned back to their initial size (p>0.05). - Papanicolaou Classes 0, III, IV and V were not found in the smears at all time points. Classes I and II were found at all time points, despite with no significant changes (p>0.05) | Both metal and ceramic brackets induce cellular alterations in the oral epithelium, despite not suggesting malignancy: in both cases the cells tend to return to their original conditions. Metal brackets caused bigger changes than ceramic brackets. |
| [43]  Buczko et al.  2017 | Prospective Observational Cohort Study aiming to assess morphological and morphometrical changes, nickel concentration and caspase-3 activation in patients undergoing fixed orthodontic treatment | - 28 patients undergoing fixed orthodontic therapy were assessed before (T0), 1 week after (T1) and 24 weeks after (T2) positioning the appliance - The smears were assessed for cytomorphometrical and cytomorphological changes and for caspase-3 activation, using under light microscopy. Nickel cellular content was assessed using graphite atomic absorption spectrometry | - N/C ration showed a significant decrease at T1 and T2, when compared to T0 - Caspase-3 activation showed a significant increase at T1, when compared with T0. The increase was not significant at T2 - Nickel cellular content showed a significant increase at T1, when compare to both T0 and T2 | Nickel released from fixed orthodontic appliances can activate caspase-3, which may be responsible for the cytotoxic action of nickel in the oral cavity |
| [44]  Carrillo-Novia et al.  2006 | Prospective Cohort Observational Study aiming to examine the use of liquid-based exfoliative cytology to investigate the presence of genomic instability and cell death in oral mucosal cells from patients fitted with metallic appliances at the initial stage of orthodontic treatment | - 18 subject receiving fixed orthodontic treatment have been assessed during the time. Three time points have been considered: before placing the orthodontic appliance (T0), 25 days (T1) and 90 days (T2) after the placement of the appliance. - The smears were assessed for the presence of MN and for Nuclear Alterations (both under light microscopy) | - T1, when compared with T0, was richer in condensed chromatin levels (p=0.01) and indicators of cell death (condensed chromatin, piknosis, karyolysis, karyorrhexis) (p=0.05). - Orthodontic patients showed higher levels of MN (p=0.04) and of nuclear buds (p=0.03) at T2, when compared to T1, indicating the presence of DNA Damage. They also showed higher levels of condensed chromatin (p=0.000) and of indicators of cells death (p=0.01) at the same time points. - The only significant difference observed between T2 and T1 is a higher karyolisis level at T2 (p=0.05) - Altri risultati qui non messi (analisi età ecc) | Orthodontic appliances can cause genomic instability and cellular death in the epithelial cells of the oral mucosa. Liquid-based cytology is an useful diagnostic tool to investigate those parameters. |
| [45]  Francis et al.  2017 | Prospective Cohort Observational Study aiming to evaluate the effects of fixed orthodontic appliances on the epithelial cells of the buccal mucosa in the form of morphometric and morphological alterations | - 30 subjects undergoing fixed orthodontic therapy were assessed at four time-points: before the beginning of the treatment (T0) and after seven (T1), thirty (T2) and forty-five (T3) days from the installation of the appliance. - The smears were assessed for the presence of MN (using light microscopy) | - The frequency of MN was significantly higher at all time-points, when compared to T0 (p<0.001). However, despite from T0 to T1 there was a great increase, the frequency of MN at T2 and T3 tended to return to T0 levels. | Fixed orthodontic appliances induce cellular alterations that are not malignant, because of the reversibility of the process. |
| [46]  Cunha et al.  2018 | Prospective Cohort Observational Study aiming to evaluate the genotoxic and cytotoxic effects of the Haas appliances in exfoliated buccal mucosa cells on patients undergoing posterior crossbite treatment | - 28 patients receiving orthodontic therapy using Haas and Hyrax appliances were assessed at three time-points: before treatment (T0), 1 month (T1) and 3 months (T2) of stabilization. - The smears were assessed for the presence of MN and Nuclear Alterations (both under light microscopy). | - Frequency of MN increased at all time-points without showing a statistically significant change (p>0.05). - Nuclear Alterations (including Pyknosis, Karyolisis, Binucleated cells and Nuclear buds) changed significantly among all time periods (p<0.0001). - Normal cells frequency decreased among all the time points, mostly between T1 and T2) (p<0.0001) | Haas appliance caused no increase in micronuclei frequency in epithelial buccal cells; however, pyknosis, karyolisis and bi-tri-/nucleated cells increased significantly |
| [47]  Gonçalves et al.  2015 | Prospective Cohort Observational Study aiming to evaluate the possible genotoxic effects of Hyrax-type expanders containing silver-soldered joints, by applying the buccal comet and micronucleus cytome assays | - 20 patients undergoing fixed orthodontic therapy are assessed for DNA damage (using the buccal comet assay), for Nuclear alterations and for frequency of MN (both using the buccal micronucleus cytome assay). The first was assessed one week before he installation of the appliance (T0) and 14 days after the installation (T1) of the appliance; the second was assessed the day of the installation (T0), 28 days (T1), 6 months (T2) and 1 year (T3) after the installation; the second was assessed | - DNA damage increased significantly between T0 and T1, as demonstrated by damage frequency increase (p=0.0071) and damage index increase (p=0.0280). - Nuclear alterations showed no significant change among all the time-points (p>0.05). - The frequency of MN did not change significantly (p=0.158), despite increasing between T0 and T1, decreasing until T2 and increasing again at 1 year after the installation (T3). | DNA damage, therefore genotoxicity effects, have been caused by silver-solder joint appliances. Nonetheless, chromosome damage did not changed significantly among the time. Future longitudinal studies should be taken. |
| [48]  Angelieri et al.  2011 | Prospective Cohort Observational Study aiming to investigate the frequency of micronucleated cells in oral mucosa from individuals who had submitted to fixed orthodontic therapy; monitor cytotoxic effects, pyknosis, karyolisis and karyorrhexis of those cells | - 23 subjects submitting to fixed orthodontic therapy have been assessed at three time-points: before the therapy (T0), during orthodontic therapy (around 170 after the installation of the appliance) (T1) and after the end of the therapy (at least 6 months after) (T2). - The smears were assessed for the presence of MN and for the presence of cellular death indicators (both using light microscopy). | - Micronuclei changed significantly along the time (p<0.05) increasing between T0 and T1, then decreasing between T1 and T2). - Indicator of cell death (namely karyorrhexis, pyknosis and karyolisis) increased non-significantly among the time-points (p>0.05) | Orthodontic therapy does not induce cytotoxic or mutagenic effects on epithelial oral cells. |
| [49]  Flores-Bracho et al.  2019 | Cross-Sectional Case-Control Observational Study aiming to analyze the micronucleus and the genotoxic effects on exfoliated cells from buccal epithelium in patients undergoing corrective orthodontic treatment at different periods | - 74 patients undergoing fixed orthodontic therapy were compared with 21 controls; the experimental group was subdivided in 4 group, based on the treatment time (T0: 1-12 months, 13-24 months, 25-48 months or more than 48 months). - The smears were assessed for the frequency of MN, for the presence of Nuclear Alterations (both under light microscopy) | - The only significant change in Nuclear Alterations was a higher level of Karyolisis in the control group, when compare to the experimental group (p=0.0166). - No significant differences were found among the sub-groups of the experimental group. | Fixed orthodontic therapy doesn’t cause genotoxic effects on the patients, independently from the treatment time. |
| [50]  Westphalen et al.  2008 | Prospective Observational Cohort Study aiming to assess DNA damage and presence of micronuclei in patients undergoing fixed orthodontic therapy before and after the placement of the appliance | - 20 patients undergoing fixed orthodontic therapy were assessed before therapy (T0) and during therapy (T1): for the comet assay T1 was 10 days after the appliance placement; fort the micronuclei assay T1 was 30 days after the placement - The smears were assessed for the presence of DNA damage and micronuclei, both under light microscopy | - The comet assay showed no significant changes between T0 and T1 - The micronuclei assay showed a significant increase in MN frequency at T1, when compared with T0 | The MN assay showed to be more sensitive than comet assay at identifying DNA damage |

Abbreviations: CA: Cytoplasmatic Area, DNA: Deoxyribonucleic Acid, MN: Micronuclei, NA: Nuclear Area, NA/CA: Nuclear/Cytoplasmatic Ratio

**Table S11**. Parameters assessed and statistical analysis performed in the studies included in this review.

| **Study** | **Parameters** | **Statistics** |
| --- | --- | --- |
| [32]  Natarajan et al.  2010 | - MN [frequency] - Cellular nickel content [ng/mL] - Cellular chromium content [ng/mL] | - Kolmogorov-Smirnov test - Mann-Whitney U test - Wilcoxon signed rank test - Spearman rank correlation coefficient |
| [33]  Hafez et al.  2011 | - Cellular nickel content [ng/mL] - Cellular chromium content [ng/mL] - Viability [%] - Composite score - Damage frequency [%] | - Levane test - Paired t-test - Wilcoxon signed rank test - Chi-square test - Fisher exact test - Simple linear regression |
| [34]  Kapadia et al.  2018 | - Cellular nickel content [ppb] - Cellular chromium content [ppb] - Cellular zinc content [ppb] - Viability [%] - Head diameter [px] - DNA in tail [%] - Tail length [%] - Tail moment [px] | - Mann-Whitney U test - Chi-square test |
| [35]  Faccioni et al.  2003 | - Cellular nickel content [ng/mL] - Cellular cobalt content [ng/mL] - Viability [%] - DNA in tail [%] - Tail length [micron] - Tail moment [px] - Damage frequency [%] | - Mann-Whitney U-test - Student t-test - Simple linear regression - Spearman r test |
| [36]  Amini et al.  2008 | - Cellular nickel content [ng/mL] - Cellular chromium content [ppb] - Cellular cobalt content [ng/mL] | - Student t-test |
| [37]  Fernández-Miñano et al. 2011 | - Cellular nickel content [microg/mL] - Cellular chromium content [microg/mL] - Cellular titanium content [microg/mL] - Cellular manganese content [microg/mL] - Cellular iron content [microg/mL] - Cellular mobyldenum content [microg/mL] - Tail moment | - Kolmogorov-Smirnov normality test - Levene test - ANOVA - Tukey test |
| [38]  Alsalhi et al.  2019 | - Cells with deviation from normal morphology [%] | - t-test |
| [39]  Sampaio Mei et al.  2013 | - NA [micron] - CA [micron] - Predominant cells [number, %] | - Kolmogorow-Smirnov test - Levene’s homogeneity test - ANOVA test - Tukey’s HSD multiple comparisons test |
| [40]  Arruda et al.  2011 | - NA [micron] - CA [micron] - Predominant cells [number, %] | - Kolmogorov-Smirnow test - Levene’s homogeneity test - ANOVA multiple comparison test - Tukey and Games-Howell test - Chi-square test |
| [41]  Rafighi et al.  2020 | - NA [micron] - CA [micron] - Predominant cells [number, %] | - Kolmogorov-Smirnov test - Shapiro Wilk test - Chi-square test - Friedman test - Wilcoxon test |
| [42]  Pereira et al.  2008 | - NA [micron] - CA [micron] - Predominant cells [number, %] | - ANOVA test - Tukey’s Honestly Significance test - McNemar’s test |
| [43]  Buczko et al.  2017 | - NA [micron] - CA [micron] - N/C | - One-way ANOVA test |
| [44]  Carrillo-Novia et al.  2006 | - MN [frequency] - NB [frequency] - CC [frequency] - KL [frequency] | - Shapiro Wilk normality test - Wilcoxon signed-rank test - Mann-Whitney U test - Kruskal-Wallis test - Chi-squared test |
| [45]  Francis et al.  2017 | - MN [frequency] | - Student t-test |
| [46]  Cunha et al.  2018 | - Normal cells [frequency] - PN [frequency] - KL [frequency] - BN [frequency] - NB [frequency] | - Analysis of variance testing - Tukey post-test - Kruskal-Wallis test - Dunn’s post-test |
| [47]  Gonçalves et al.  2015 | - Normal cells [frequency] - MN [frequency] - PN [frequency] - KL [frequency] - BN [frequency] - NB [frequency] - Damage frequency [%] - Damage index | - Shapiro-Wilk test - Wilcoxon test (BCA) - Linear Mixed Model Analysis (BMCyt) |
| [48]  Angelieri et al.  2011 | - Normal cells [frequency] - MN [frequency] - PN [frequency] - KL [frequency] - KR [frequency] | - Friedman test |
| [49]  Flores-Bracho et al.  2019 | - MN - BN - PN - KL - NB   All of the parameters were expressed as [median, confidence limits for free distribution – 95%] | - Anderson-Darling test - Chi-square test - ANOVA - Kruskal-Wallis test - Dunn’s post-test - Fisher’s exact test - Mann-Whitney test |
| [50]  Westphalen et al.  2008 | - NA [micron] - CA [micron] - Damage index | - t-test - Fisher exact test |

Abbreviations: CA: Cytoplasmatic Area, CC: Condensed Chromtin, DNA: Deoxyribonucleic Acid, KL: Karyolysis, KR: Karyorrhexis, MN: Micronuclei, NA: Nuclear Area, N/C: Nuclear/Cytoplasmatic Ratio, NB: Nuclear Buds, PK: Pyknosis

**References**

1. Baricevic M, Mravak-Stipetic M, Majstorovic M, Baranovic M, Baricevic D, Loncar B. Oral mucosal lesions during orthodontic treatment. Int J Paediatr Dent. 2011 Mar;21(2):96-102. doi: 10.1111/j.1365-263X.2010.01078.x. Epub 2010 Dec 2. PMID: 21121986.
2. Kvam E, Gjerdet NR, Bondevik O. Traumatic ulcers and pain during orthodontic treatment. Community Dent Oral Epidemiol. 1987 Apr;15(2):104-7. doi: 10.1111/j.1600-0528.1987.tb00493.x. PMID: 3471374.
3. Jomova K, Valko M. Advances in metal-induced oxidative stress and human disease. Toxicology. 2011 May 10;283(2-3):65-87. doi: 10.1016/j.tox.2011.03.001. Epub 2011 Mar 23. PMID: 21414382.
4. Dressler VL, Müller EI, Pozebon D. Bioimaging Metallomics. Adv Exp Med Biol. 2018;1055:139-181. doi: 10.1007/978-3-319-90143-5_7. PMID: 29884965.
5. Lin Y, Gross ML. Mass Spectrometry-Based Structural Proteomics for Metal Ion/Protein Binding Studies. Biomolecules. 2022 Jan 15;12(1):135. doi: 10.3390/biom12010135. PMID: 35053283; PMCID: PMC8773722.
6. Mikulewicz M, Chojnacka K, Woźniak B, Downarowicz P. Release of metal ions from orthodontic appliances: an in vitro study. Biol Trace Elem Res. 2012 May;146(2):272-80. doi: 10.1007/s12011-011-9233-4. Epub 2011 Oct 20. PMID: 22011837; PMCID: PMC3310133.
7. Pereira T, Kesarkar K, Tamgadge A, Bhalerao S, Shetty S. Comparative analysis of oral rinse-based cytology and conventional exfoliative cytology: A pilot study. J Cancer Res Ther. 2018 Jul-Sep;14(5):921-925. doi: 10.4103/0973-1482.179095. PMID: 30197326.
8. Cervix cancer screening. IARC handbooks of cancer prevention. Vol. 10. Lyon, France: International Agency for Research on Cancer, 2005 (https://publications.iarc.fr/380).
9. Malacarne IT, Takeshita WM, de Souza DV, Dos Anjos Rosario B, de Barros Viana M, Renno ACM, Salvadori DMF, Ribeiro DA. Is micronucleus assay in oral exfoliated cells a useful biomarker for biomonitoring populations exposed to pesticides? A systematic review with meta-analysis. Environ Sci Pollut Res Int. 2022 Sep;29(43):64392-64403. doi: 10.1007/s11356-022-22015-x. Epub 2022 Jul 19. PMID: 35854069.
10. Strober W. Trypan Blue Exclusion Test of Cell Viability. Curr Protoc Immunol. 2015 Nov 2;111:A3.B.1-A3.B.3. doi: 10.1002/0471142735.ima03bs111. PMID: 26529666; PMCID: PMC6716531.
11. Collins AR. Measuring oxidative damage to DNA and its repair with the comet assay. Biochim Biophys Acta. 2014 Feb;1840(2):794-800. doi: 10.1016/j.bbagen.2013.04.022. Epub 2013 Apr 22. PMID: 23618695.
12. Kuchařová M, Hronek M, Rybáková K, Zadák Z, Štětina R, Josková V, Patková A. Comet assay and its use for evaluating oxidative DNA damage in some pathological states. Physiol Res. 2019 Mar 6;68(1):1-15. doi: 10.33549/physiolres.933901. Epub 2018 Oct 23. PMID: 30433808.
13. Martín-Cameán A, Jos Á, Mellado-García P, Iglesias-Linares A, Solano E, Cameán AM. In vitro and in vivo evidence of the cytotoxic and genotoxic effects of metal ions released by orthodontic appliances: A review. Environ Toxicol Pharmacol. 2015 Jul;40(1):86-113. doi: 10.1016/j.etap.2015.05.007. Epub 2015 May 21. PMID: 26093195.
14. Downarowicz P, Mikulewicz M. Trace metal ions release from fixed orthodontic appliances and DNA damage in oral mucosa cells by in vivo studies: A literature review. Adv Clin Exp Med. 2017 Oct;26(7):1155-1162. doi: 10.17219/acem/65726. PMID: 29211366.
15. Piñeda-Zayas A, Menendez Lopez-Mateos L, Palma-Fernández JC, Iglesias-Linares A. Assessment of metal ion accumulation in oral mucosa cells of patients with fixed orthodontic treatment and cellular DNA damage: a systematic review. Crit Rev Toxicol. 2021 Aug;51(7):622-633. Doi: 10.1080/10408444.2021.1960271. Epub 2021 Nov 5. PMID: 34738508.
16. Aromataris, E.; Munn, Z. JBI Manual for Evidence Synthesis. 2020. Available online: https://synthesismanual.jbi.global (accessed on 16 September 2023).
17. Page M.J., McKenzie J.E., Bossuyt P.M., Boutron I., Hoffmann T.C., Mulrow C.D., Shamseer L., Tetzlaff J.M., Akl E.A., Brennan S.E., Chou R., Glanville J., Grimshaw J.M., Hróbjartsson A., Lalu M.M., Li T., Loder E.W., Mayo-Wilson E., McDonald S., McGuinness L.A., Stewart L.A., Thomas J., Tricco A.C., Welch V.A., Whiting P., Moher D. The PRISMA 2020 statement: an updated guideline for reporting systematic reviews. BMJ. 2021, 372, 71.
18. Cohen J. Weighted kappa: nominal scale agreement with provision for scaled disagreement or partial credit. Psychol Bull. 1968 Oct;70(4):213-20. doi: 10.1037/h0026256. PMID: 19673146.
19. Fleiss JL: Statistical Methods for Rates and Proportions (ed 2). New York, Wiley, 1981, pp. 38-46
20. National Heart, Lung, and Blood Institute. Study Quality Assessment Tool. Available online: https://www.nhlbi.nih.gov/health-topics/study-quality-assessment-tools (accessed on 12 January 2024).
21. Gonçalves e Silva CR, Oliveira LD, Leão MV, Jorge AO. Candida spp. adherence to oral epithelial cells and levels of IgA in children with orthodontic appliances. Braz Oral Res. 2014;28:28-32. doi: 10.1590/s1806-83242013005000031. PMID: 25000599.
22. Low B, Lee W, Seneviratne CJ, Samaranayake LP, Hägg U. Ultrastructure and morphology of biofilms on thermoplastic orthodontic appliances in 'fast' and 'slow' plaque formers. Eur J Orthod. 2011 Oct;33(5):577-83. doi: 10.1093/ejo/cjq126. Epub 2010 Dec 27. PMID: 21187528.
23. Leung NM, Chen R, Rudney JD. Oral bacteria in plaque and invading buccal cells of young orthodontic patients. Am J Orthod Dentofacial Orthop. 2006 Dec;130(6):698.e11-8. doi: 10.1016/j.ajodo.2006.05.028. PMID: 17169727.
24. Faccioni P, De Santis D, Sinigaglia S, Pancera P, Faccioni F, Nocini PF. Short-term "in vivo" study on cellular DNA damage induced by acrylic Andresen activator in oral mucosa cells. Orthod Craniofac Res. 2019 Aug;22(3):208-212. doi: 10.1111/ocr.12312. Epub 2019 Apr 11. PMID: 30908883.
25. Cruz JPP, Santos NCND, Pithon MM, de Morais Marcílio Cerqueira E. Biomonitoring of children and adolescents using orthodontic appliances made of acrylic resins through micronucleus testing of exfoliated buccal and palatal mucosa cells. Am J Orthod Dentofacial Orthop. 2021 Aug;160(2):193-199. doi: 10.1016/j.ajodo.2020.03.037. Epub 2021 May 8. PMID: 33975748.
26. Dallel I, Ben Salem I, Merghni A, Bellalah W, Neffati F, Tobji S, Mastouri M, Ben Amor A. Influence of orthodontic appliance type on salivary parameters during treatment. Angle Orthod. 2020 Jul 1;90(4):532-538. doi: 10.2319/082919-562.1. PMID: 33378497; PMCID: PMC8028469.
27. Raducanu, A.-M.; Mihai, S.; Sandu, I.; Anghel, A.; Furnica, C.; Chistol, R.O.; Dinu, C.A.; Tutunaru, D.; Earar, K. Quantification of Salivary Nitric Oxide in Patients with Fixed Orthodontic Treatment. Appl. Sci. 2022, 12, 8565. https://doi.org/10.3390/app12178565
28. Guler C, Toy E, Ozturk F, Gunes D, Karabulut AB, Otlu O. Evaluation of salivary total oxidant-antioxidant status and DNA damage of children undergoing fixed orthodontic therapy. Angle Orthod. 2015 Mar;85(2):239-44. doi: 10.2319/110113-798.1. Epub 2014 Jun 20. PMID: 24949910; PMCID: PMC8631878.
29. Angelieri F, Carlin V, Saez DM, Pozzi R, Ribeiro DA. Mutagenicity and cytotoxicity assessment in patients undergoing orthodontic radiographs. Dentomaxillofac Radiol. 2010 Oct;39(7):437-40. doi: 10.1259/dmfr/24791952. PMID: 20841462; PMCID: PMC3520189.
30. Taubmann A, Willershausen I, Walter C, Al-Maawi S, Kaina B, Gölz L. Genotoxic and cytotoxic potential of methacrylate-based orthodontic adhesives. Clin Oral Investig. 2021 May;25(5):2569-2581. doi: 10.1007/s00784-020-03569-x. Epub 2020 Sep 24. PMID: 32970196; PMCID: PMC8060203.
31. Durgo K, Orešić S, Rinčić Mlinarić M, Fiket Ž, Jurešić GČ. Toxicity of Metal Ions Released from a Fixed Orthodontic Appliance to Gastrointestinal Tract Cell Lines. Int J Mol Sci. 2023 Jun 9;24(12):9940. doi: 10.3390/ijms24129940. PMID: 37373088; PMCID: PMC10297913.
32. Natarajan M, Padmanabhan S, Chitharanjan A, Narasimhan M. Evaluation of the genotoxic effects of fixed appliances on oral mucosal cells and the relationship to nickel and chromium concentrations: an in-vivo study. Am J Orthod Dentofacial Orthop. 2011 Sep;140(3):383-8. doi: 10.1016/j.ajodo.2010.07.027. PMID: 21889083.
33. Hafez HS, Selim EM, Kamel Eid FH, Tawfik WA, Al-Ashkar EA, Mostafa YA. Cytotoxicity, genotoxicity, and metal release in patients with fixed orthodontic appliances: a longitudinal in-vivo study. Am J Orthod Dentofacial Orthop. 2011 Sep;140(3):298-308. doi: 10.1016/j.ajodo.2010.05.025. PMID: 21889074.
34. Kapadia JM, Agarwal AR, Mishra S, Joneja P, Yusuf AS, Choudhary DS. Cytotoxic and Genotoxic effect on the Buccal Mucosa Cells of Patients Undergoing Fixed Orthodontic Treatment. J Contemp Dent Pract. 2018 Nov 1;19(11):1358-1362. PMID: 30602641.
35. Faccioni F, Franceschetti P, Cerpelloni M, Fracasso ME. In vivo study on metal release from fixed orthodontic appliances and DNA damage in oral mucosa cells. Am J Orthod Dentofacial Orthop. 2003 Dec;124(6):687-93; discussion 693-4. doi: 10.1016/j.ajodo.2003.09.010. PMID: 14666083.
36. Amini F, Borzabadi Farahani A, Jafari A, Rabbani M. In vivo study of metal content of oral mucosa cells in patients with and without fixed orthodontic appliances. Orthod Craniofac Res. 2008 Feb;11(1):51-6. doi: 10.1111/j.1601-6343.2008.00414.x. PMID: 18199080.
37. Fernández-Miñano E, Ortiz C, Vicente A, Calvo Guirado JL, Ortiz AJ. Metallic ion content and damage to the DNA in oral mucosa cells of children with fixed orthodontic appliances. Biometals. 2011 Oct;24(5):935. doi: 10.1007/s10534-011-9448-z. Epub 2011 Apr 6. Erratum in: Biometals. 2018 May 3;: PMID: 21468621.
38. Alsalhi R, Alkhedhairi L, Alsaikhan S, Bilal R, Ghneim S. Epithelial Cells in Patients with Fixed Orthodontic Appliances. J Coll Physicians Surg Pak. 2019 Oct;29(10):1012-1014. doi: 10.29271/jcpsp.2019.10.1012. PMID: 31564282.
39. Sampaio Mei RM, Soares de Lima AA, Filho JB, Tanaka OM, Filho OG, Camargo ES. A cytological analysis of the oral mucosa adjacent to orthodontic devices. Eur J Gen Dent 2013;2:119-23.
40. Arruda EP, Trevilatto PC, Camargo ES, Woyceichoski IE, Machado MA, Vieira I, Lima AA. Preclinical alterations of oral epithelial cells in contact with orthodontic appliances. Biomed Pap Med Fac Univ Palacky Olomouc Czech Repub. 2011 Sep;155(3):299-303. doi: 10.5507/bp.2011.043. PMID: 22286817.
41. Rafighi A, Sohrabi A, Zokaee M, Moghaddam SF, Sharghi R. Evaluation of the epithelial cells of lower lip mucosa after debonding of fixed orthodontic appliances. Minerva Stomatol. 2020 Aug;69(4):245-250. doi: 10.23736/S0026-4970.19.04238-9. PMID: 32945633.
42. Pereira BR, Tanaka OM, Lima AA, Guariza-Filho O, Maruo H, Camargo ES. Metal and ceramic bracket effects on human buccal mucosa epithelial cells. Angle Orthod. 2009 Mar;79(2):373-9. doi: 10.2319/021508-92.1. PMID: 19216594.
43. Buczko P, Szarmach I, Grycz M, Kasacka I. Caspase-3 as an important factor in the early cytotoxic effect of nickel on oral mucosa cells in patients treated orthodontically. Folia Histochem Cytobiol. 2017;55(1):37-42. doi: 10.5603/FHC.a2017.0004. PMID: 28509315.
44. Carrillo-Novia I, Lara-Carrillo E, Torres-Bugarin O, Morales-Valenzuela AA, Salmerón-Valdés EN, Hegazy-Hassan W, Velázquez-Enríquez U, Toral-Rizo VH. Use of liquid-based cytology samples reveals genomic instability and cell death in patients undergoing orthodontic treatment. J Oral Sci. 2023 Jan 11;65(1):24-28. doi: 10.2334/josnusd.22-0235. Epub 2022 Dec 16. PMID: 36529513.
45. Francis PG, Thomas M, Antony V, Shaloob M, Hassan KJ, Roshan G. Cytomorphometric Analysis on the Effects of Components of OrthodonticAppliances on the Epithelial Cells of the Buccal Mucosa. J Int Soc Prev Community Dent. 2017 May-Jun;7(3):142-146. doi: 10.4103/jispcd.JISPCD_58_17. Epub 2017 May 22. PMID: 28584785; PMCID: PMC5452568.
46. Cunha AS, Castillo WO, Takahashi CS, Küchler EC, Segato RAB, da Silva LAB, Romano FL, Matsumoto MAN, Nelson-Filho P. Genotoxic and cytotoxic effects of Haas appliance in exfoliated buccal mucosa cells during orthodontic treatment. Angle Orthod. 2018 Sep;88(5):590-595. doi: 10.2319/101117-687.1. Epub 2018 May 15. PMID: 29761706; PMCID: PMC8183133.
47. Gonçalves TS, Menezes LM, Trindade C, Thomas P, Fenechc M, Henriques JA. In vivo evaluation of the genotoxic effects of Hyrax auxiliary orthodontic appliances containing silver-soldered joints. Mutat Res Genet Toxicol Environ Mutagen. 2015 Sep;791:25-9. doi: 10.1016/j.mrgentox.2015.07.007. Epub 2015 Jul 23. PMID: 26338539.
48. Angelieri F, Carlin V, Martins RA, Ribeiro DA. Biomonitoring of mutagenicity and cytotoxicity in patients undergoing fixed orthodontic therapy. Am J Orthod Dentofacial Orthop. 2011 Apr;139(4 Suppl):e399-404. doi: 10.1016/j.ajodo.2009.06.029. PMID: 21435548.
49. Flores-Bracho MG, Takahashi CS, Castillo WO, Saraiva MCP, Küchler EC, Matsumoto MAN, Ferreira JTL, Nelson-Filho P, Romano FL. Genotoxic effects in oral mucosal cells caused by the use of orthodontic fixed appliances in patients after short and long periods of treatment. Clin Oral Investig. 2019 Jul;23(7):2913-2919. doi: 10.1007/s00784-018-02795-8. Epub 2019 Jan 5. PMID: 30612244.
50. Westphalen GH, Menezes LM, Prá D, Garcia GG, Schmitt VM, Henriques JA, Medina-Silva R. In vivo determination of genotoxicity induced by metals from orthodontic appliances using micronucleus and comet assays. Genet Mol Res. 2008;7(4):1259-66. doi: 10.4238/vol7-4gmr508. PMID: 19065761.
51. Igelström E, Campbell M, Craig P, Katikireddi SV. Cochrane's risk of bias tool for non-randomized studies (ROBINS-I) is frequently misapplied: A methodological systematic review. J Clin Epidemiol. 2021 Dec;140:22-32. doi: 10.1016/j.jclinepi.2021.08.022. Epub 2021 Aug 23. PMID: 34437948; PMCID: PMC8809341.
